# Cryptic surface-associated multicellularity emerges through cell adhesion and its regulation

**DOI:** 10.1371/journal.pbio.3001250

**Published:** 2021-05-13

**Authors:** Jordi van Gestel, Andreas Wagner

**Affiliations:** 1 Department of Evolutionary Biology and Environmental Studies, University of Zürich, Zürich, Switzerland; 2 Swiss Institute of Bioinformatics, Lausanne, Switzerland; 3 The Santa Fe Institute, Santa Fe, New Mexico, United States of America; Institute of Science and Technology Austria (IST Austria), AUSTRIA

## Abstract

The repeated evolution of multicellularity led to a wide diversity of organisms, many of which are sessile, including land plants, many fungi, and colonial animals. Sessile organisms adhere to a surface for most of their lives, where they grow and compete for space. Despite the prevalence of surface-associated multicellularity, little is known about its evolutionary origin. Here, we introduce a novel theoretical approach, based on spatial lineage tracking of cells, to study this origin. We show that multicellularity can rapidly evolve from two widespread cellular properties: cell adhesion and the regulatory control of adhesion. By evolving adhesion, cells attach to a surface, where they spontaneously give rise to primitive cell collectives that differ in size, life span, and mode of propagation. Selection in favor of large collectives increases the fraction of adhesive cells until a surface becomes fully occupied. Through kin recognition, collectives then evolve a central-peripheral polarity in cell adhesion that supports a division of labor between cells and profoundly impacts growth. Despite this spatial organization, nascent collectives remain cryptic, lack well-defined boundaries, and would require experimental lineage tracking technologies for their identification. Our results suggest that cryptic multicellularity could readily evolve and originate well before multicellular individuals become morphologically evident.

## Abstract 2

This modelling study reveals that cell adhesion can lead to the cryptic origination of surface-associated multicellularity, where collectives cannot be distinguished by eye but nonetheless express emergent multicellular adaptations.

## Introduction

**Fig 1:**
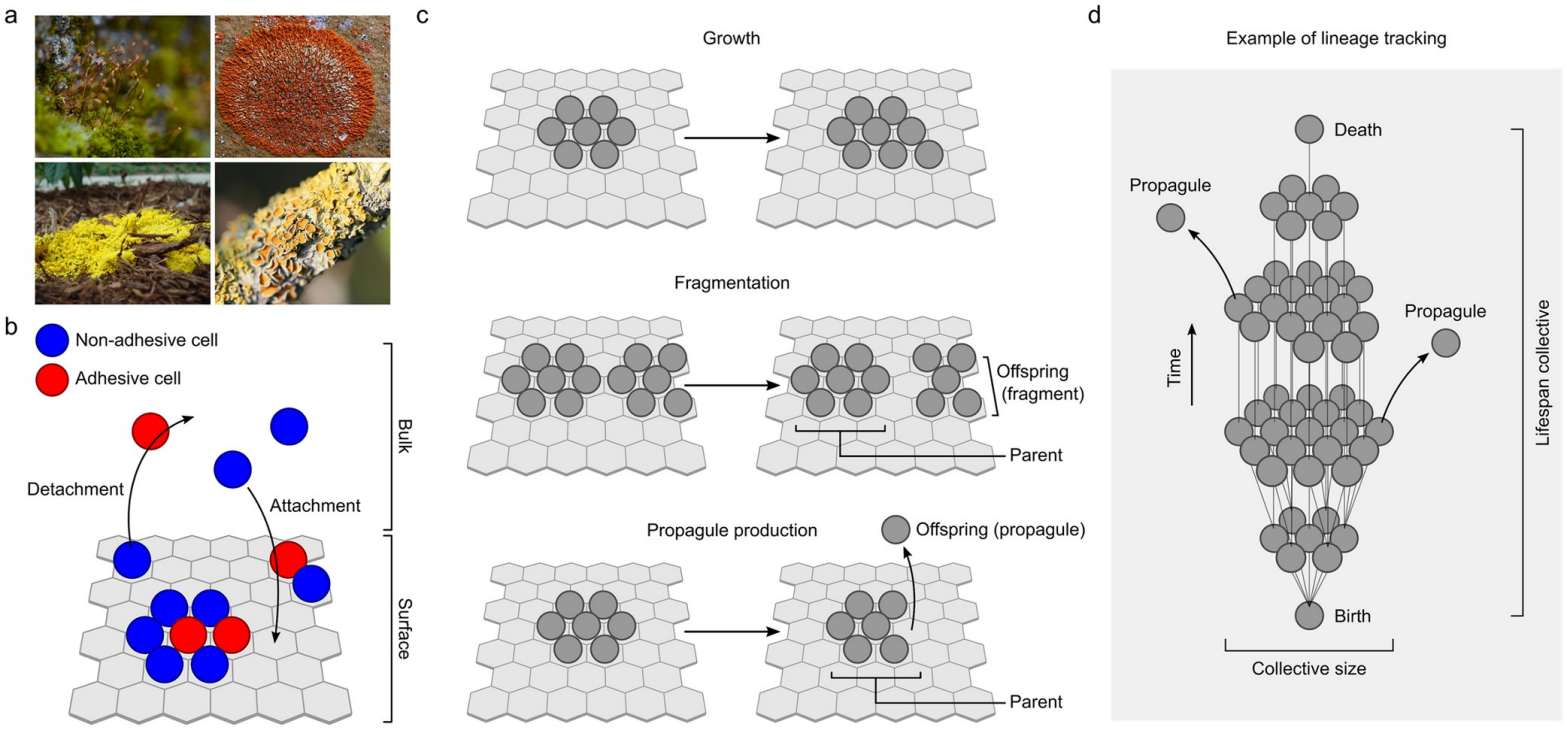
Surface colonization and tracking cell collectives. (a) Examples of surface-associated multicellularity, top–bottom: Bryophyta, Ascomycota, slime mold, and lichen (images are shown under the Creative Commons license from Wikimedia and Needpix; Rostislav Kralik, Jason Hollinger). (b) Schematic depiction of model adopted from [41]. Cells can occupy 2 environments: the surface and the bulk. In the bulk, cells freely move around. The surface is represented by a hexagonal grid to which cells can attach. Adhesive cells (red) can adhere to the surface independently. Nonadhesive cells (blue) can only adhere when neighboring an adhesive cell. (c) Processes of collective growth and collective reproduction. Collective reproduction can either result from fragmentation of the collective on the surface or from propagule production, where a cell detaches to the bulk. (d) Simplified depiction of lineage tracking from “birth” to “death” of a collective. Collective size is determined by the number of cells in the collective. Collective life span is determined by the number of time steps during which the collective exists.

Surface-associated organisms can be found throughout the biosphere [1–3]. By adhering to surfaces, organisms can obtain primary access to resources important for growth. Lichens, for instance, adhere to rocks to obtain access to sunlight, and microbes colonize our teeth to consume sugars (Fig 1a). In competition for space, organisms evolved suites of multicellular adaptations that promote surface growth or dispersal, like filamentous growth, sporangia, and fruiting bodies. This raises the question how surface-associated multicellularity comes about in evolutionary time. One important challenge in studying surface-associated organisms resides in their remarkable plasticity [4–7]: On a surface, organisms often display continuous growth, can merge, and adopt many different forms. This plasticity makes it difficult to apply standard evolutionary theory, like multilevel selection theory [8–10] or kin selection theory [11,12], to study how surface-associated multicellularity originates [13]. Here, we overcome this challenge by introducing a novel bottom-up approach, based on spatial lineage tracking of cells, to study how primitive cell collectives can first emerge on a surface and subsequently evolve multicellular adaptations.

The life cycle of surface-associated organisms involves both surface growth and reproduction. Surface growth relies on adhesion. Organisms express a wide variety of adhesive molecules, from extracellular polysaccharides secreted into the environment [14–17] to membrane-bound proteins that mediate cell-to-cell contact [18,19]. These molecules ensure that cells remain attached while dividing. Within a multicellular collective, cells often express diverse adhesive properties and thereby affect the spatial organization of the collective [20,21]. For example, in the soil bacterium *Bacillus subtilis*, cells differentiate into adhesive and nonadhesive cells that together promote colony expansion [21,22]. To bring about collective organization, cells can also directly interact with each other using so-called kin recognition systems, such as strain-specific cell–cell communication [23–25]. These systems generally promote cooperation between cells and thereby foster the emergence of collectivity [26]. For instance, in many surface-associated pathogens, cells employ cell-to-cell communication to collectively produce virulence factors, which are costly to express but promote infectious growth [27].

Besides growth, reproduction forms another major component in the life cycle of surface-associated organisms. A multicellular collective reproduces whenever cells disassociate from the existing collective, with the potential of reestablishing a collective somewhere else. Collective reproduction therefore differs from cell division, as it concerns propagation of the collective and not that of the individual cell. In contrast to surface growth, which relies on adhesion, collective reproduction depends on a loss of adhesion and can take several forms [28,29]. For example, when adhesion weakens, individual cells can disassociate from the collective one by one, disperse, and colonize new surfaces. Such cells are typically referred to as propagules. By regulating adhesion, organisms can control the rate of propagule production [30–32]. For example, inside bacterial biofilms, cells can facilitate dispersal by down-regulating polysaccharide production or actively secreting enzymes that degrade polysaccharides [33,34]. Collectives can also break apart into multiple smaller collectives through a process called fragmentation [35]. In many species, including fungi, myxobacteria, and slime molds, reproduction occurs through the formation of specialized dispersal organs, called fruiting bodies, in which cells collectively rise from the surface to facilitate spore dispersal [36–38].

A critical step in understanding the emergence and subsequent evolution of multicellularity lies in understanding how changes in cell properties affect collective processes like growth and reproduction. What collective phenotypes emerge spontaneously and what other phenotypes are attainable through mutations? To address these questions from a theoretical perspective, one needs a bottom-up approach that explicitly accounts for cell properties and examines how, through the evolution of those properties, collectives emerge and evolve [31,39,40]. This approach is blind to the exact form of multicellularity that may eventually arise but instead aims at understanding the causal processes that lead to multicellular organization. Here, we model the evolution of surface colonization using such a bottom-up approach and thereby study the earliest emergence of surface-associated multicellularity [41].

Our model emulates an ecology where cells can occupy two possible environments: a surface or the bulk—the three-dimensional space above the surface (Fig 1b). For simplicity, the surface is represented by a two-dimensional hexagonal grid, where each cell can have up to 6 neighbors (see S1 Text for a discussion on alternative surface geometries). Surface attachment requires adhesion. Since adhesion is known to also mediate cell–cell contact [20,42,43], we assume that, for attachment, cells must either be adhesive themselves or associate with an adhesive cell on the surface. Thus, adhesive cells can support the attachment of nonadhesive neighbors. Detachment results from a loss of adhesion. It requires that both the detaching cell and its neighbors are nonadhesive.

In the bulk, cells do not adhere to each other and strongly compete. That is, we assume that cells in the bulk can only divide when the population size is below a given carrying capacity. Cells can escape this competition by attaching to the surface, where they can divide whenever there is space in one of the neighboring grid elements (see Methods). In other words, the surface forms a distinct ecological niche, where cells only compete for space with other surface-associated cells and not with cells from the bulk. After cell division on the surface, a daughter cell can have two possible fates: It can either remain attached to the surface through adhesion, or it can detach to the bulk (for details, see S1 Text). In many surface colonizers, adhesion requires the expression of costly molecules [42,44,45], and we thus assume that adhesion lowers the rate of cell division. When competition for space on the surface increases, because more cells attach to the surface, the costs of adhesion can outweigh its benefits (i.e., the opportunity to divide on the surface). We start by assuming that adhesion is expressed stochastically, as observed in several colony-forming bacteria [46,47].

In order to keep track of collectives that emerge on the surface, we follow their constituent cells in time [48–50], akin to methods of lineage tracking employed in both developmental biology [51] and microbiology [52] (Fig 1c and 1d). We monitor instances of (i) cell division; (ii) cell death; (iii) surface attachment; (iv) surface detachment; and (v) changes in cell adhesion (S2 Text, S1 and S2 Figs). A surface-associated collective is seeded when a cell attaches to the surface. On the surface, this collective can grow (Fig 1c). Every time a cell divides, and its daughter cell remains attached to the surface, the collective grows by one cell. A collective reproduces when its constituent cells get disconnected, either through fragmentation or propagule production (Fig 1c). During fragmentation, formerly connected cells on the surface get disconnected because a constituent cell dies or detaches. We define the largest fragment as the parental collective and the other fragment(s) as its offspring. Propagule production occurs when single cells detach from the surface. This could either results from a loss of adhesion or from cell division (when the daughter cell detaches). Finally, we assume that cells experience a constant death rate. This implies that collectives can also die and that large collectives are less likely to die than small collectives. By tracking how a collective grows, reproduces, and dies, we can reconstruct its genealogy (S2 Fig). This allows us to examine various collective properties (Fig 1d), including spatial organization, number of offspring, and life span.

Importantly, to make sure that we account for all cells when reconstructing collective genealogies, we also consider “collectives” consisting of single cells only. For example, if a collective produces a propagule, through the detachment of a single cell from the surface, this single cell is counted as an independent collective. This implies that all cells in the bulk are traced as single cell “collectives.” Also, when collectives happen to merge on the surface, as is frequently observed in primitive surface colonizers, we still trace their constituent cells separately, so that we can monitor each collective from the first cell that seeds the collective to the last cell that dies.

## Results

### Surface colonization

**Fig 2:**
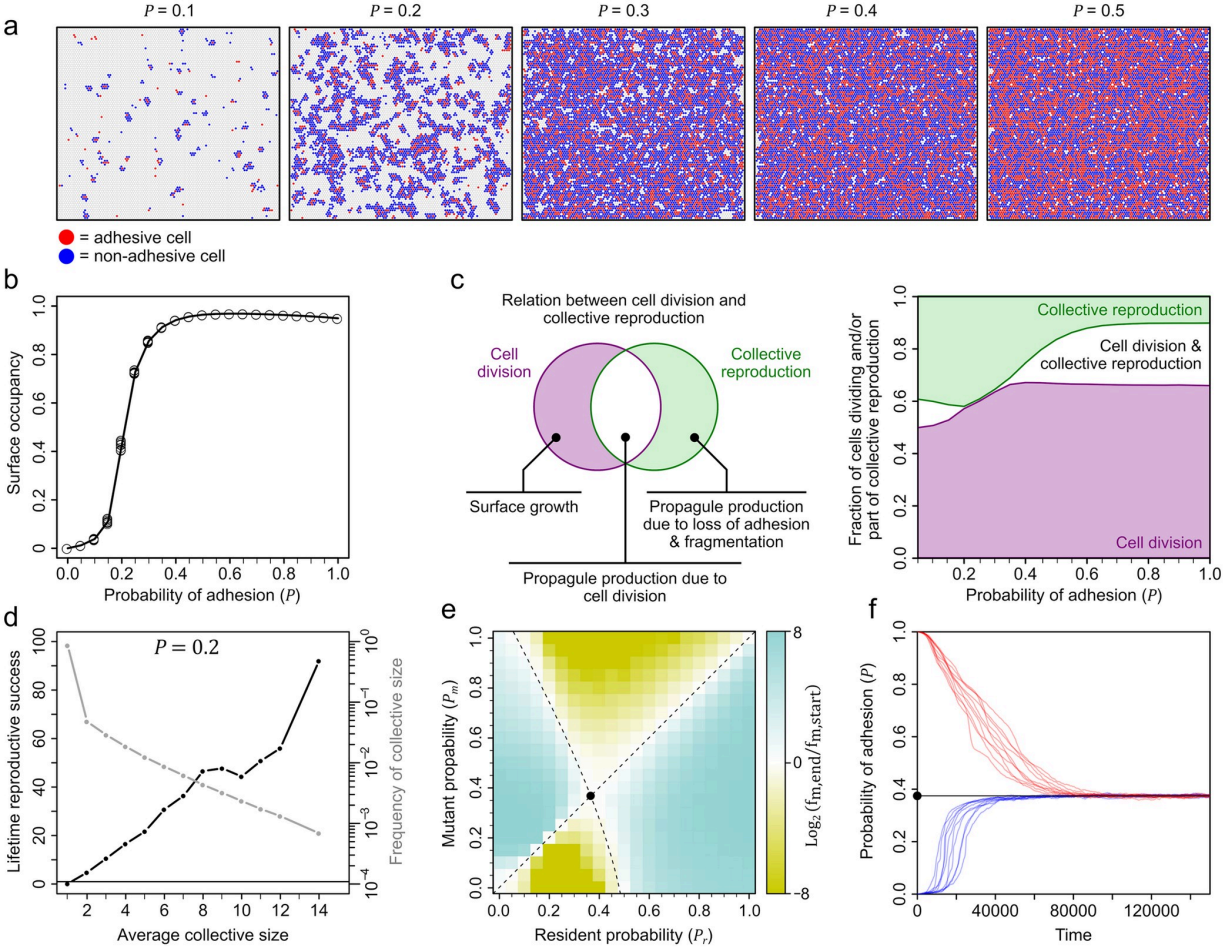
Relation between adhesion probability and collective properties. (a) Surface with adhesive (red) and nonadhesive (blue) cells at different adhesion probabilities. (b) Relation between surface occupancy and adhesion probability (*n* = 10). (c) Left, Venn diagram showing the conceptual relationship between cell division and collective reproduction: purple, cell division without collective reproduction, which results in collective growth; white, cell division that leads to detaching propagule; green, collective reproduction without cell division (e.g., fragmentation or propagule production due to loss of adhesion). (c) Right, fraction of cells on the surface that undergo cell division and/or are part of collective reproduction. (d) Relation between lifetime reproductive success and collective size (i.e., number of cells) for *P* = 0.2. (e) Pairwise invasibility analysis (*n* = 10, for every competition of resident and mutant genotypes): white, fitness of resident and mutant genotypes are the same; cyan, fitness of mutant genotype is higher; ocre, fitness of resident genotype higher (see S1 Text). Black dot, adhesion probability (P*) favored in competition. Dashed lines show isoclines where resident and mutant genotypes are equally fit. (f) Evolution of adhesion probability in populations starting from *P* = 0 (blue lines; *n* = 10) and *P* = 1 (red lines; *n* = 10). Black vertical line: P*. Raw data are provided in S2 Data in Github repository https://github.com/jordivangestel/PLoS-Biology-2021.

We start our analysis by examining how the probability (*P*) of adhesion affects the organization of cells on the surface. As expected, the number of cells adhering to the surface increases with the adhesion probability (Fig 2a and 2b; see also S1–S10 Movies and S2 Fig). When *P* > 0.35, the surface becomes fully occupied.

On the surface, cell division can either lead to collective growth when a daughter cell remains attached, or to reproduction, when the daughter cell detaches and thus forms a propagule of the collective (S1 Fig). To determine how cell division contributes to collective reproduction, we monitor all instances of both cell division and collective reproduction on the surface. Irrespective of adhesion probability, we find that most cell division events lead to surface growth (Fig 2c). Propagule production only occurs at low and high adhesion probabilities. Specifically, when the adhesion probability is low (*P* < 0.2), cell division often leads to propagule production because newly dividing cells are likely to detach. The reason is that adhesive cells to which they could remain attached are scarce on the surface. Conversely, when the adhesion probability is high (*P* > 0.35), cell division often leads to propagule production due to a lack of space on the surface. At intermediate adhesion probabilities (0.2 < *P* < 0.35), division exclusively contributes to collective growth (Fig 2c). In this regime, collectives reproduce either through fragmentation or through propagules that detach due to a loss of adhesion. Thus, depending on the propensity to adhere, collectives display different modes of reproduction to which cell division contributes either directly or indirectly, via growth.

Next, we trace collectives from birth till death (Fig 1d, S2 Fig). This allows us to determine a collective’s lifetime reproductive success, which is the total number of offspring it produces during its lifetime (S2 Fig). When the overall population size reaches equilibrium, the average lifetime reproductive success of collectives is expected to be approximately one, meaning that each collective on average produces a single offspring. We start by tracking all cell collectives at an adhesion probability of *P* = 0.2 (Fig 2d). This reveals that lifetime reproductive success strongly depends on collective size. Most collectives are small, consisting of a single cell only (86%), and have an average lifetime reproductive success of 0.13 offspring. In contrast, the largest collectives (consisting of 14 cells on average) are rare (0.06%) but have an enormous lifetime reproductive success of 92 offspring. This strong reproductive skew entails that large collectives contribute a disproportional number of offspring to future generations. Most of this offspring consists of single-cell propagules that are short lived and fail to reproduce. Higher adhesion probabilities increase the fraction of large collectives, but simultaneously lower their reproductive output, due to the costs of adhesion (S3 Fig).

Thus, our model predicts that merely by varying the propensity to adhere, cells can give rise to diverse collectives that differ in size (Fig 2d, S2 and S3 Figs), mode of reproduction (Fig 2c), and lifetime reproductive success (Fig 2d and S3 Fig). These properties spontaneously arise through the interaction of cells on the surface and do not require any form of regulation, such as the expression of adhesion in response to collective size, as seen in many colony-forming bacteria [53]. Moreover, because collectives congregate on the surface, their properties are largely cryptic and cannot be distinguished by visually inspecting the surface at any moment in time. Only through lineage tracking can we identify individual collectives and study their properties [48].

### Evolution of adhesion

So far, we studied how the adhesion probability affects the emergence of cell collectives on the surface. Next, we turn to the question how the adhesion probability itself might evolve. Given the strong reproductive skew in favor of large collectives, we predict that selection would favor higher adhesion probabilities, which lead to larger collectives, until the costs of adhesion no longer outweigh the benefits of growth. To find out, we allowed adhesion to vary by mutation in individual cells, as, for example, observed in many bacterial species [43,54–56]. We subsequently performed pairwise competitions between multiple combinations of mutant and resident genotypes, where a mutant has an adhesion probability of *P*_*m*_ and the resident of *P*_*r*_. At the onset of competition, we assume that 10% of cells carry the mutant genotype and all cells occur in the bulk. We then determined whether the mutant increases in frequency during surface competition with the resident genotype. Fig 2e shows the results of all pairwise competitions, which reveals that selection favors an adhesion probability of P* ≈ 0.4. Lower adhesion probabilities (*P* < P*) are selected against, because they result in smaller collectives (when *P*_*r*_ < P*, *P*_*m*_ > *P*_*r*_ can invade; Fig 2e and S3 Fig) and larger adhesion probabilities (*P* > P*) are selected against, because they increase the cost of adhesion without increasing the collective size, due to a lack of space (when *P*_*r*_ > P*, *P*_*m*_ < *P*_*r*_ can invade; Fig 2e and S3 Fig).

To confirm that P* is indeed favored by selection, we also performed evolutionary simulations. In these simulations, we start with a population of only adhesive cells (*P* = 1) or no adhesive cells (*P* = 0) and assume that dividing cells have a small probability of incurring a mutation that slightly alters the adhesion probability (S1 Text). We find that populations always converge to the same adhesion probability of P* = 0.37 ± 0.004 (mean ± SD; *n* = 100) regardless of the initial conditions, which supports our pairwise competition analysis (Fig 2f). The exact adhesion probability that evolves, P*, does depend on the variables such as the cost of adhesion. When adhesion cost increases, P* decreases (S4 Fig).

In sum, our model predicts that surface colonization can rapidly evolve through mutations that affect the propensity to adhere. This prediction is corroborated by numerous laboratory experiments. For example, within days, *Pseudomonas fluorescence* cells can colonize the air-liquid interface, due to mutations that lead to the overproduction of an adhesive cellulosic polymer [54,57]. Similarly, within tens of generations, *B*. *subtilis* colonies can accumulate mutations that alter the fraction of adhesive cells in the population [58]. The ease by which adhesion evolves also has clinical implications [59]. For instance, during chronic lung infection of cystic fibrosis patients, *Pseudomonas aeruginosa* rapidly evolves mucoid colonies [55,60]. These colonies overproduce the adhesive polysaccharide alginate, which enhances surfaces attachment and increases antibiotic resistance [61]. Taken together, these studies emphasize the strong selective benefit of surface growth.

### Evolution of regulation

Until now, we ignored the role of regulation in the evolution of surface colonization. Regulation, however, plays a critical role in the evolution of multicellularity, because it enables cells to coordinate their properties to the benefit of collective growth or reproduction [20,62–65]. Accordingly, in many surface-associated organisms, cells regulate the spatiotemporal expression of adhesion [18,66,67]. Cells can, for instance, tune the expression of adhesion depending on the adhesive molecules they sense in the collective, using so-called adhesion receptors [68]. Cells can also directly sense other cells within the collective, using so-called kin recognition systems, and adjust their adhesive properties in response [69,70]. Even primitive surface colonizers can have elaborate signal transduction cascades to control the expression of adhesion [71]. For example, in the bacterium *B*. *subtilis*, colony formation depends on communicative signals [72,73], osmotic stress [74], adhesive molecules [68], and nutrient stress [71]. Regulation affects when and where in a colony cells express adhesion and thereby also affects colony growth [21,22].

**Fig 3:**
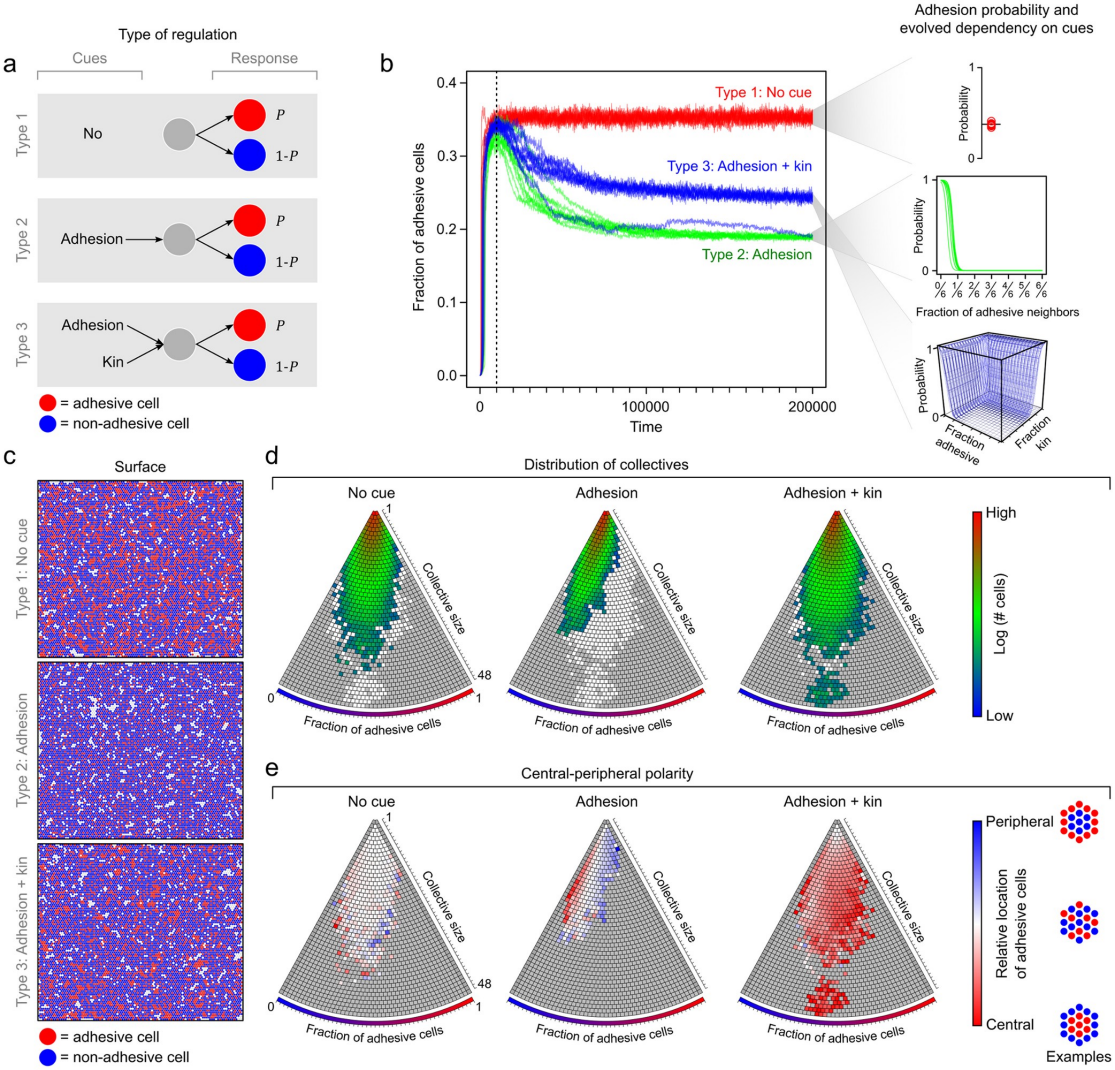
Type of regulation profoundly impacts evolution of cell collectives. (a) Schematic depiction of 3 idealized types of regulation. Cues that cells sense and the probability that cells become adhesive (*P*, red) or nonadhesive (1 − *P*, blue). (b) Left, fraction of adhesive cells in population over evolutionary time (*n* = 10; see also S6 Fig): red, type 1; green, type 2; blue, type 3. (b) Right, evolved dependency of adhesion probability on environmental cues (*n* = 10). (c) Surface at the end of evolution. (d) Distribution of collectives according to their size and the fraction of adhesive cells within the collective: grey, collectives that are never observed; white, collectives that are observed in other regulatory systems; color, log-transformed number of cells that are associated with observed collectives. (e) Central-peripheral polarity in cell adhesion: red, adhesive cells are localized closer to center than nonadhesive cells; blue, nonadhesive cells are localized closer to center than nonadhesive cells; white, no difference between localization of adhesive and nonadhesive cells. Note, since localization of adhesive cells is determined relative to that of nonadhesive cells, polarity can only be established for collective with both adhesive and nonadhesive cells.

In this section, we examine how regulation impacts the evolution of surface colonization. For our purpose, we compare 3 idealized types of regulation, which we detail below (Fig 3a). In each type, we start with a population of nonadhesive cells and subsequently explore how the fraction of adhesive cells evolves in time. In addition, we examine how collectives are organized at the end of evolution, by quantifying (i) their size; (ii) their fraction of adhesive cells; and (iii) the spatial arrangement of those cells.

When adhesion is expressed stochastically and is not subject to regulation (type 1), we observe a rapid increase in the fraction of adhesive cells over evolutionary time, until the surface becomes fully occupied. The fraction of adhesive cells stabilizes to approximately one-third of the population (0.35 ± 0.006; mean ± SD, *n* = 100; Fig 3b and 3c, S11 Movie). Collectives are strongly skewed in size, such that most consist of few cells (Fig 3d), and they show no signs of spatial organization (Fig 3e). That is, adhesive cells are not preferentially localized towards either the center or the periphery of collectives.

In modeling regulation, we follow experimental evidence by assuming that cells can detect adhesive molecules and regulate their own adhesion in response [68] (type 2). In many surface-associated organisms, cells use membrane-bound receptors to sense adhesive molecules [75–77]. These receptors can either bind to the adhesive molecules directly [68] or sense them indirectly through, for example, changes in the osmolarity [74]. To emulate these regulatory mechanisms, we assume that cells can increase or decrease the adhesion probability (*P*) in response to the fraction of adhesive neighbors (see Methods). We also assume that cells do not express adhesion at the onset of evolution. During evolution, they can accrue mutations that affect the adhesion probability (*P*) and its dependency on the fraction of adhesive neighbors by either increasing or decreasing *P*.

We first examine how regulation affects the fraction of adhesive cells and its change in evolutionary time. As in the absence of regulation, the fraction of adhesive cells initially increases, but then after a surface becomes fully occupied, the fraction steadily declines to a much lower value of approximately 0.19 ± 0.002 (*n* = 100) at the end of evolution. The decline in adhesive cells can be explained by the gradual evolution of a regulatory switch (Fig 3b), where cells become adhesive (*P* ≈ 1) in the absence of adhesive neighbors and become nonadhesive (*P* ≈ 0) otherwise. This switch assures that cells remain attached to the surface, while avoiding the costs of adhesion, because they only become adhesive when strictly necessary (i.e., in the absence of adhesive neighbors). When there are no costs associated with the expression of adhesion, the regulatory switch does not evolve (S4 Fig). The regulatory switch also affects the spatial arrangement of cells within a collective (Fig 3e): If the fraction of adhesive cells is higher than average, they are localized towards the periphery of the collective, and when the fraction of adhesive cells is lower than average, they are localized towards the center. This spatial arrangement is concordant with a checkerboard pattern, where adhesive cells are equally spaced out over the surface (Fig 3c, S7 Fig, and S12 Movie), as, for example, observed during epithelia development [78]. Thus, the regulation of adhesion strongly impacts both the fraction of adhesive cells inside a collective as well as their spatial arrangement. In contrast, in the absence of regulation, no such spatial organization can evolve, because cells have no information about their neighboring cells.

In addition to sensing adhesive molecules, we also examine the impact of kin recognition (type 3). Surface-associated organisms express a plethora of kin recognition mechanisms, from strain-specific communication systems to interlocking membrane-bound receptors [24,69,70,79–83]. These systems enable cells to sense kin, which—in the case of clonal growth—consist of closely related or genetically identical cells. Kin recognition systems can profoundly impact the organization of collectives, by facilitating cooperative interactions between cells [84]. For example, in many bacterial colonies, cells communicate by secreting diffusible quorum-sensing molecules whose concentration varies with colony size, which allows cells to respond to the colony size by for example inducing dispersal [53,85,86].

We here consider an idealized kin recognition system, where cells can sense the fraction of neighboring cells with the same genotype, thereby emulating a membrane-bound recognition system [69,87]. We define a cell’s genotype by its regulatory response to its surrounding cells, such that cells from the same kin group have the same adhesion probability in any given environment. We do not address how kin recognition itself evolves, which has intensively been studied elsewhere [88–91], but instead explore how the presence of kin recognition, as observed in many surface-associated organisms, could potentially affect the evolution of collectivity. We implement kin recognition in addition to adhesion regulation, meaning that cells can sense both the fraction of adhesive cells and of kin in their neighborhood and either increase or decrease the adhesion probability in response (Methods).

Fig 3b shows how the fraction of adhesive cells changes over evolutionary time in this scenario. The fraction of adhesive cells first steeply increases and then slowly declines until, at the end of evolution, 0.25 ± 0.058 (*n* = 100) of the cells are adhesive, which is intermediate to the fraction of adhesive cells observed without regulation and with adhesion regulation alone ($\chi_{\left(2\right)}^{2}=257.11$, *p* < 10^−16^). In contrast to the other regulatory systems, the fraction of adhesive cells varies with the collective size: Small collectives have fewer adhesive cells than large collectives (S5 Fig; see also S13 Movie). This size-dependent adhesion can be explained by the evolved regulatory response (Fig 3b): Cells become adhesive (*P* ≈ 1) when they have no adhesive neighbors or when they are surrounded by kin. Since cells in large collectives are more likely to be surrounded by kin than those in small collective, the fraction of adhesive cells increases with collective size, which mimics quorum-sensing responses seen in several bacterial species [53]. Intriguingly, kin recognition also leads to spatial polarity. Cells in the center of collectives are more likely to be surrounded by kin than those at the edge, which results in a central-peripheral polarity, in which adhesive cells are localized towards the center (Fig 3e).

The central-peripheral polarity strongly benefits collective growth: Adhesive cells in the center of the collective support the surrounding nonadhesive cells in staying attached to the surface. By avoiding the costs of adhesion, these nonadhesive cells have a competitive advantage in surface competition with neighboring collectives. Being surrounded by kin, the adhesive cells in the center are largely shielded from such competition (S7 Fig). Thus, even though the central-peripheral polarity increases the overall fraction of adhesive cells within a collective, and thereby the costs, it benefits surface growth by fostering a division of labor between adhesive and nonadhesive cells. The profound impact of this spatial organization on surface growth is perhaps best illustrated in a small fraction of the simulations (approximately 3%), where growth is so effective that one genotype monopolizes nearly the entire surface, causing a massive collapse in genetic diversity and a steep increase in the overall fraction of adhesive cells (S6 Fig).

Besides analyzing the properties of collectives at the end of evolution, we also examined their growth dynamics in time (S3 Text). Irrespective of the type of regulation, we observe that collectives undergo diverse conformational changes in time, affecting both their size and form (S3 Text, S8–S12 Figs). In contrast to these diverse growth dynamics, reproduction mostly occurs through single-cell propagules (S13 Fig). These results broadly mimic the growth patterns of primitive surface colonizers in nature, where growth opportunities on the surface determine the exact size and form of collectives, but where collectives nevertheless express a consistent spatial organization, like the central-peripheral polarity observed in this study [33,36,92,93].

In summary, our model predicts that regulation of adhesion can strongly impact collective growth. Specifically, we observe that the evolution of a central-peripheral polarity in cell adhesion fosters the division of labor between adhesive and nonadhesive cells. In *B*. *subtilis*, a very similar division of labor has been observed [21,94]: Cells in the colony center produce a costly molecule, surfactin, that promotes surface growth of cells at the periphery. Like in our model, where the central-peripheral polarity depends on kin recognition, surfactin production is triggered by a kin-specific quorum-sensing signal [26,95,96]. We postulate that central-peripheral polarity may form a generic organizing principle that profits surface growth, because it allows cells at the border to specialize in surface competition, while being supported by cells in the center. For that reason, it might not be surprising that kin recognition systems are widely observed in surface-associated organisms, from primitive colony-forming bacteria like *B*. *subtilis* to colonial animals [24,69,70,79–81].

## Discussion

**Fig 4:**
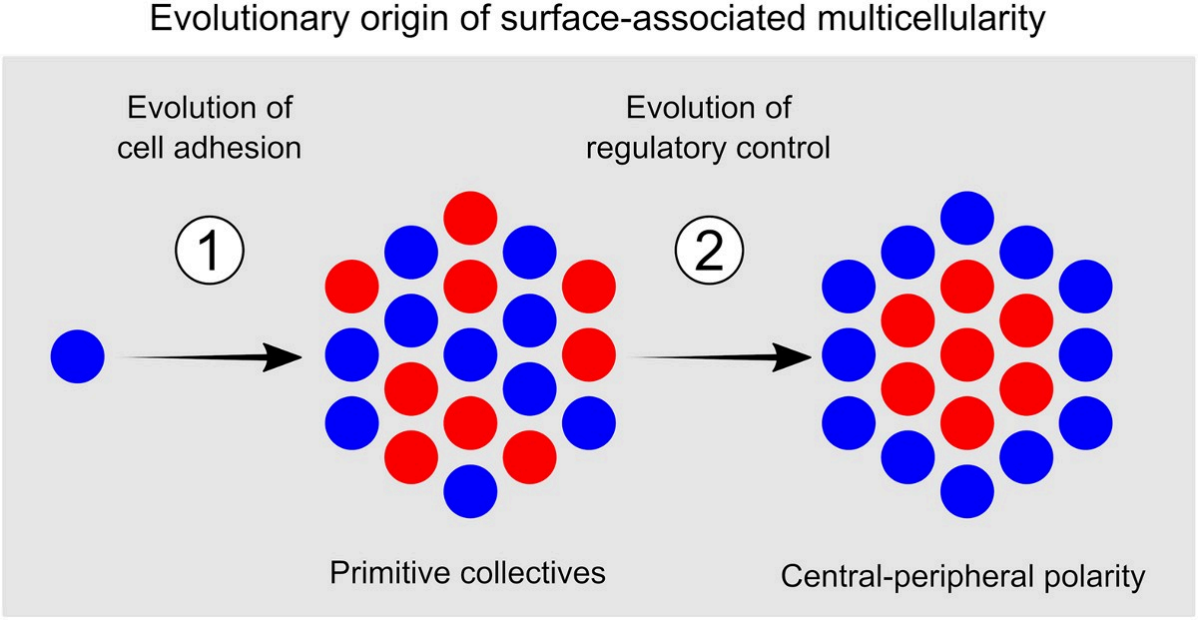
Proposed evolutionary origin of surface-associated multicellularity. First, cell adhesion evolves, leading to cryptic cell collectives on a surface. Second, regulation evolves which leads to central-peripheral polarity, which brings about a characteristic collective organization.

In this study, we showed how a primitive form of surface-associated multicellularity can readily evolve in the presence of two commonly observed cell-level properties: cell adhesion and the regulatory control of adhesion. By merely evolving the propensity to adhere, cells rapidly colonize a surface, where they form primitive cell collectives (step 1 in Fig 4). These first collectives show no consistent spatial organization and closely resemble forms of bacterial surface colonization that can spontaneously evolve in the lab [43,54,60]. Collectives nevertheless express diverse properties, differing in size, mode of propagation, and lifetime reproductive success. A strong reproductive skew in favor of large collectives drives the evolution of adhesion. Then, through kin recognition, collectives evolve a central-peripheral polarity in cell adhesion (step 2 in Fig 4). This simple multicellular organization profoundly impacts surface growth by facilitating a division of labor between adhesive and nonadhesive cells (S6 Fig).

Throughout their evolution, collectives retain a largely cryptic appearance (see Fig 3c). That is, when visually inspecting a surface, one would not be able to distinguish between neighboring collectives, because there are no morphological boundaries that separate them. Lineage tracking is the only means to identify and examine such primitive surface-associated collectives. Our modeling approach contrasts with previous models on the evolution of multicellularity, which did not apply lineage tracking. For instance, many evolutionary models start by assuming the presence of physically separated collectives. These models do not address how collectives emerge, but instead ask how—after their origination—collectives evolve properties important for multicellular organization, such as cooperation [9,97,98] or division of labor [99–101]. Similarly, many developmental models consider collectives in isolation and ask how cells can give rise to collective properties through self-organization [40,102–104]. Finally, there is a large group of models that, like ours, study how cells interact and evolve on a surface [105–109]. As cells divide and die on a surface, they spontaneously give rise to collectives, but instead of tracking these collectives, most models revert their attention to the underlying genotypes and ask what genotypes prevail over evolutionary time [105–109]. As a result, it often remains unclear how the earliest surface-associated cell collectives are organized and how this organization subsequently affects a collective’s competitive success in terms of surface growth and reproduction. In contrast, our model makes some concrete predictions about emergent cell collectives. For instance, it predicts that, at least initially, collectives are strongly skewed in size, with a few large collectives producing most single-cell propagules. In addition, our model predicts that a central-peripheral polarity in cell adhesion can result in a strong competitive advantage, sometimes leading to a few genotypes monopolizing the entire surface. An exciting task for future work is to validate these predictions experimentally.

Theoretical models on surface growth are paralleled by a large body of empirical literature where surface-associated organisms are studied by either growing collectives in isolation (e.g., biofilms, colonies, pellicles, swarms, fruiting bodies) [110–113], thereby focusing on their multicellular organization [36,114–118], or by competing several genotypes against each other on a surface [30,119–124]. Competition experiments revealed that spatial interactions can strongly affect the outcome of competition, oftentimes leading to the coexistence of multiple genotypes [43,120,125]. However, due to technical challenges and the transient nature of many primitive cell collectives [126], there is often a limited quantitative understanding of how genotypes exactly give rise to individual cell collectives and how these collectives subsequently grow and propagate in competition with other collectives. Since the spatiotemporal organization of collectives determines a genotype’s competitive success (i.e., rates of collective growth and reproduction)—making collectives the relevant unit of biological organization [49,50]—we need to understand how collective organization comes about to uncover how primitive forms of surface-associated multicellularity first arise and subsequent evolve.

Fortunately, over the last decade, methods of empirical lineage tracking have strongly improved through advances in single-cell microscopy [126–128], image analysis [129], single-cell sequencing [51,130,131], and genome editing [132,133], thereby opening up the possibility of studying primitive cell collectives across a wide range of organisms [126]. For example, the stochastic and combinatorial expression of fluorescent proteins makes it possible to trace up to approximately 100 lineages without interrupting the spatial configuration of cells [134]. Moreover, recent developments combining barcoding with fluorescence in situ hybridization have increased the spatial resolution at which cell lineage can be traced [135] and dual-view light sheet microscopy has made it possible to track entire cell populations in real time without interruption [127,136]. Finally, advances in spatial transcriptomics make it possible to simultaneously examine the expression of hundreds of genes in space, which, for instance, revealed that bacteria cells can express vastly distinct expression profiles within the same colony [137]. Currently, most of the above technologies are applied to study a few genetically tractable model organisms, but they hold the promise of uncovering many still hidden forms of multicellular organization in nonmodel organisms as well.

Cryptic origins of biological organization exist on all spatiotemporal scales. On the smallest scale, the spatial organization of an embryo is often laid out through an asymmetric distribution of patterning molecules in a developing oocyte, long before embryonic development creates any visible spatial organization [138,139]. On the largest scale, reproductive isolation often exists in cryptic species with little or no apparent morphological or ecological differences [140,141]. Suitable experimental technologies are essential to reveal such hidden differences and understand the evolutionary origins of crypticity [140]. To identify cryptic multicellularity, these technologies involve state-of-the-art lineage tracking methods, which can uncover a hidden diversity of surface-associated multicellularity, both primitive and advanced. Given how few and widely met prerequisites our model needs to create multicellular organization, we expect that cryptic multicellularity will be widespread and that it will originate long before the first multicellular individuals become morphologically evident.

## Methods

We study the early emergence of surface-associated multicellularity using an individual-based model, which builds on a previous model of surface colonization [41]. The model emulates an ecology where cells can occupy 2 possible environments: the bulk and the surface. The bulk is a three-dimensional space above the surface (e.g., water column), where cells can freely move around but cannot adhere to each other. Cells can attach to the surface through adhesion. The surface is represented by a hexagonal grid of 100 × 100 grid elements (see S1 Text of an detailed discussion on alternative surface geometries). Each grid element can be occupied by a single cell, which can have up to 6 neighboring cells. In total, the surface can therefore be colonized by 10,000 cells.

In the model, we only account for cell-level events and use spatial lineage tracking (see S2 Text) to determine how collectives spontaneously emerge on the surface. Cells can express 2 distinct phenotypes, adhesive or nonadhesive, and can undergo 1 of 5 possible events at every timestep: (1) surface attachment; (2) surface detachment; (3) cell division; (4) cell death; and (5) phenotypic change in adhesion. S1 Text details each of these events. In short, cells can attach to a surface when they are adhesive or associate with an adhesive cell on the surface. We thereby assume that adhesion mediates both cell–surface and cell–cell contact. Cells detach when they are nonadhesive and not associated with an adhesive neighbor. Cell division depends on both the environment in which cells occur (surface versus bulk) and their phenotype (adhesive versus nonadhesive). In the bulk, cells can only divide when the population size is below a predetermined carrying capacity (*K*_*bulk*_ = 5,000 cells). On the surface, cells can divide whenever there is space in one of the neighboring grid elements. After division on the surface, a cell could remain attached or it could immediately detach to the bulk. Because adhesion is known to be costly, we assume that adhesive cells have lower cell division rate than nonadhesive cells, irrespective of the environment. In our evolutionary simulations, cells have a small probability of incurring a mutation at every cell division (for details, see S1 Text). Cell death occurs with a fixed probability, irrespective of the environment and phenotype of cells. Finally, cells can change their phenotype: With a probability *P*, a cell becomes or remains adhesive, and with a probability 1 − *P*, a cell becomes or remains nonadhesive. Depending on the simulations, the adhesion probability (*P*) is either unchanging (Fig 2a–2e) or subject to evolutionary change (Figs 2f and 3). In addition, depending on the type of regulation (Fig 3), the adhesion probability can depend on the fraction of adhesive cells and fraction of kin (see S1 Text for details on the adhesion probability).

At the onset of the simulations, we assume that cells occur in the bulk and that none of them is adhesive. Unless specified otherwise, we perform nonevolutionary simulations (Fig 2), where cells have a fixed adhesion probability, for 1,000 timesteps (*T*_*max*_), which equals approximately 20 cell generations. We perform evolutionary simulations (Figs 2f and 3) for 200,000 timesteps, which equals approximately 4,000 cell generations. For further parameter values, see S1 Text. Since the model only accounts for cell-level events, we monitor collectives by following their constituent cells in time. In this way, we can determine how collectives first appear on a surface through cell attachment and subsequently grow and reproduce. We measure various collective properties, including collective size, spatial organization, and life span. In S2 Text, we specify how each cell-level event can affect the processes of collective growth and reproduction. In short, collective growth results from cell division on the surface, where a daughter cell remains surface-attached next to the mother cell. Collective reproduction can result from cell division, surface detachment, or cell death. Cell division and surface detachment result in propagule production, where a (daughter) cell that detaches to the bulk forms a propagule of the collective on the surface. Surface detachment and cell death can lead to fragmentation, where a collective of formerly connected cells splits up in 2 or more smaller collectives.

## Supporting information

S1 Fig Relation between cell-level events and collective reproduction and growth. (a) Scenario 1: cell death leading to collective reproduction through fragmentation. Cell death (yellow) can lead to the fragmentation of 2 groups of previously connected cells. (b) Scenario 2: cell detachment leading to reproduction. The detaching cell forms a propagule that originates from within a collective. Detachment can also lead to fragmentation of 2 groups of previously connected cells. (c) Scenario 3: cell division leading to collective reproduction. On a surface, cell division can lead to propagule production when a daughter cell does not remain attached. In the bulk, cells are never associated, so cell division by definition results in reproduction. Note that in the bulk, cells are counted as single cell “collectives.” (d) Scenario 4: cell division leading to collective growth when a daughter cell remains attached to the surface.
(TIF)

S2 Fig Surface colonization and cell collectives. (a) Surface at end of simulations with adhesive (red) and nonadhesive (blue) cells at *P* = [0.1,1]. (b) Representative cell collectives traced from birth till death (see also Fig 1d): lines, cell lineage underlying cell collective showing both adhesive (red) and nonadhesive (blue) cells; branches, cell division; light green dots, collective offspring resulting from fragmentation; dark green dots, collective offspring resulting from propagule production; green convex hull illustrates relative size of collective. Offspring are not traced in time. Cell lineages show relative location of cells within the collective.
(TIF)

S3 Fig Lifetime reproductive success increases with collective size. Lifetime reproductive success (left *y* axis) is determined by the total number of offspring resulting from either propagule production or fragmentation (Fig 1c), produced over the life span of a collective. Average collective size (*x* axis) refers to the average number of cells that constitute a collective over the life span of the collective. Frequency of collectives (right *y* axis) with given size. Blue, purple, and red, probability (*P*) that cells express adhesion of respectively 0.2, 0.4, and 0.6. Raw data are provided in S2 Data in Github repository https://github.com/jordivangestel/PLoS-Biology-2021.
(TIF)

S4 Fig Reaction norms that evolved at different adhesion costs. Reaction norms (*n* = 5) of most abundant genotypes at the end of evolution for different costs of adhesion, where the cost of adhesion is defined by the cell division rate of adhesive cells relative to that of nonadhesive cells. Each reaction norm shows the probability of adhesion (*P*) as a function of both the fraction of adhesive cells and the fraction of kin in a cell’s neighborhood (i.e., the 6 neighboring positions surrounding a cell on the hexagonal surface). Irrespective of the type of regulation, when the costs of adhesion increase (i.e., lower cell division rate of adhesive cells), the probability of adhesion decreases (i.e., reaction norms color less red and more blue). Within each type of regulation, the shape of the reaction norm remains qualitatively the same for a wide range of adhesion costs. Top-bottom, reaction norms associated with type 1, 2, and 3 regulation.
(TIF)

S5 Fig Relation between fraction of adhesive cell and collective size. Average fraction of adhesive cells in collectives of different size for type 1 (red), 2 (green), and 3 (blue) regulation. Only in type 3 regulation there is a strong increase in the fraction of adhesive cells when collectives increase in size, while there is only a weak increase for the other types of regulation. Raw data are provided in S2 Data in Github repository https://github.com/jordivangestel/PLoS-Biology-2021.
(TIF)

S6 Fig Evolution of type 3 regulation leads to 3 alternative evolutionary outcomes. (a) Average fraction of adhesive cells in 100 independent simulations of different type of regulation: red, no cue; green, adhesion only regulation; red, adhesion regulation + kin recognition. (b and c) Detailed analysis of simulations in type 3 regulation. In type 3 simulations, we observe 3 alternative evolutionary outcomes, where fraction of adhesive cells on the surface is (outcome 1, light blue) low, (outcome 2, blue) intermediate, or (outcome 3, dark blue) high. In outcome 1 (3/100 simulations; light blue), kin recognition does not evolve; cells therefore behave the same as those in adhesion only regulation (type 2), which results in a low fraction of adhesive cells on the surface (b). In outcome 2 (94/100, blue), kin recognition evolves, and cells behave as described in the main text (Fig 3), resulting in an intermediate fraction of adhesive cells (b). In outcome 3 (3/100), kin recognition evolves and leads to the dominance of one genotype on the surface. This results in a high fraction of adhesive cells because most cells are surrounded by kin (b). Since one genotype monopolizes the surface, it also results in a collapse of the genotypic diversity in the population, as shown by a strong reduction in the Shannon diversity shown in panel c.
(TIF)

S7 Fig Clusters of interconnective adhesive cells on the surface are most similar in type 3 regulation. (a) Examination of the diversity underlying clusters of interconnected adhesive cells (directly neighboring each other) on the surface at the end of evolution. For each cluster, we analyze cells one by one and determine their genotype and the collectives to which they belong. Based on this, we calculate the underlying Shannon diversity (*H* = −∑*p*_*i*_ ⋅ ln *p*_*i*_). When all cells in the cluster have the same genotype, the Shannon diversity of genotypes is 0. By the same token, when all cells in the cluster belong to the same collective, the Shannon diversity of collectives is 0. When the Shannon diversity of collectives is higher than 0, it means that the clusters of interconnected cells consists of multiple collectives that merged on the surface. Boxplots in (a) show median Shannon diversity (horizontal black line), interquartile range (box), data within 1.5 times interquartile range above the upper quartile and below the lower quartile (whiskers) and outliers. The diversity analysis uncovers two main findings: First, clusters of interconnected adhesive cells are most homogeneous in type 3 regulation, meaning that they more often consist of cells with the same genotype and cells belonging to the same collective. Thus, in the presence of kin recognition, adhesive cells are more likely to be surrounded by kin than in the absence of kin recognition. Second, for all types of regulation and clusters sizes, the diversity of genotypes is lower than the diversity of collectives, meaning that clusters often consist of multiple collectives that merged on the surface but have the same genotype (hence increasing the diversity of collectives underlying a cluster but not the diversity of genotypes). (b) Surface at the end of evolution for different types of regulation, revealing clusters of interconnected adhesive cells on the surface (i.e., clusters of red cells). Raw data are provided in S2 Data in Github repository https://github.com/jordivangestel/PLoS-Biology-2021.
(TIF)

S8 Fig Procedure for identifying spatial configurations. First, all collectives are extracted from the surface, as indicated by the different numbers. Second, the spatial configuration of each collective is determined by examining the spatial location of both adhesive (red) and nonadhesive cells (blue) within the collective. Third, the spatial configurations are compared between collectives to identify all unique configurations, thereby correcting for radial symmetry. This results in a list of unique spatial configurations together with the number of collectives that display each spatial configuration.
(TIF)

S9 Fig Spatial configuration of collectives can be characterized by their size and fraction of adhesive cells. (a) Each node corresponds to a unique spatial configuration that is expressed by at least 100 of 5 ⋅ 10^7^ analyzed collectives. The distance between two nodes is determined by their similarity (see Methods). The node size corresponds to the number of collectives that express the associated spatial configuration. (b) Collective size of different spatial configurations. (c) Fraction of adhesive cells in spatial configuration.
(TIF)

S10 Fig Collectives from different types of regulation express different spatial configurations. For each spatial configuration (i.e., node in the network), we determine how frequently it is observed among collectives from each type of regulation. This reveals that small collectives with many adhesive cells are more commonly observed in type 1 regulation (a, red), collectives with low fraction of adhesive cells are more commonly observed in type 2 regulation (b, green) and large collectives are more commonly observed in type 3 regulation (c, blue).
(TIF)

S11 Fig Type of regulation affects how collectives change their spatial configuration in time. Transparent nodes show unique spatial configurations, like shown in S9 and S10 Figs. Connections show how collectives change their spatial configuration in time (see also S12 Fig for individual examples). Color shows frequency by which change in spatial configuration is observed in different types of regulation: red, changes mostly observed in type 1 regulation; green, changes mostly observed in type 2 regulation; blue, changes mostly observed in type 3 regulation.
(TIF)

S12 Fig Examples of how collectives change their spatial configuration in time from their origin till the moment they first reproduce. Each panel shows an example of how a collective in one of the 3 regulatory systems changes its spatial configuration in time. In each example, the collective is seeded by a nonadhesive cell, which grows on the surface and thereby gives rise to different spatial configurations. Upper, bold black lines show within the network representation how a collective changes its spatial configuration in time. Below, small representations of collective show how the spatial configuration of the collective changes in time. We trace each collective until the first successful reproductive event that leads to a nonadhesive propagule (small grey arrow), thereby completing the life cycle. (a) Example of how collective changes its spatial configuration in the type 1 regulation. (b) Example of how collective changes its spatial configuration in the type 2 regulation. (c) Example of how collective changes its spatial configuration in the type 3 regulation.
(TIF)

S13 Fig Most offspring collectives are nonadhesive single-cell propagules. (a) Lines connect spatial configuration of parental collectives and offspring collectives upon reproduction. (b) Red nodes highlight spatial configuration of offspring collectives. The frequency by which a configuration is observed is indicated by the saturation of the fill color. Most offspring collectives have the same spatial configuration: nonadhesive single-cell propagules (see also S9 Fig).
(TIF)

S1 Movie Surface colonization with 10% probability of expressing adhesion. (MP4)

S2 Movie Surface colonization with 20% probability of expressing adhesion. (MP4)

S3 Movie Surface colonization with 30% probability of expressing adhesion. (MP4)

S4 Movie Surface colonization with 40% probability of expressing adhesion. (MP4)

S5 Movie Surface colonization with 50% probability of expressing adhesion. (MP4)

S6 Movie Surface colonization with 60% probability of expressing adhesion. (MP4)

S7 Movie Surface colonization with 70% probability of expressing adhesion. (MP4)

S8 Movie Surface colonization with 80% probability of expressing adhesion. (MP4)

S9 Movie Surface colonization with 90% probability of expressing adhesion. (MP4)

S10 Movie Surface colonization with 100% probability of expressing adhesion. (MP4)

S11 Movie Surface colonization in evolved type 1 regulation. (MP4)

S12 Movie Surface colonization in evolved type 2 regulation. (MP4)

S13 Movie Surface colonization in evolved type 3 regulation. (MP4)

S1 Text Model. Description of model, which includes a description of all cell level events, determination of the adhesion probability for the 3 different types of regulation, pseudocode, simulation conditions, and a discussion on the role of the surface geometry.
(PDF)

S2 Text Spatial lineage tracking. Description of collective growth, reproduction, and death.
(PDF)

S3 Text Growth dynamics of collectives. Analysis of growth dynamics of collectives.
(PDF)

S1 Data C++ code available from the Github repository https://github.com/jordivangestel/PLoS-Biology-2021. (ZIP)

S2 Data Raw data of summary figures available from the Github repository https://github.com/jordivangestel/PLoS-Biology-2021. (XLSX)
