# Supplementary figures and images for: Cryptic surface-associated multicellularity emerges through cell adhesion and its regulation
Source: PLoS Biol. 2021 May 13;19(5):e3001250. doi: 10.1371/journal.pbio.3001250 (PMC8148357; doi:10.1371/journal.pbio.3001250)

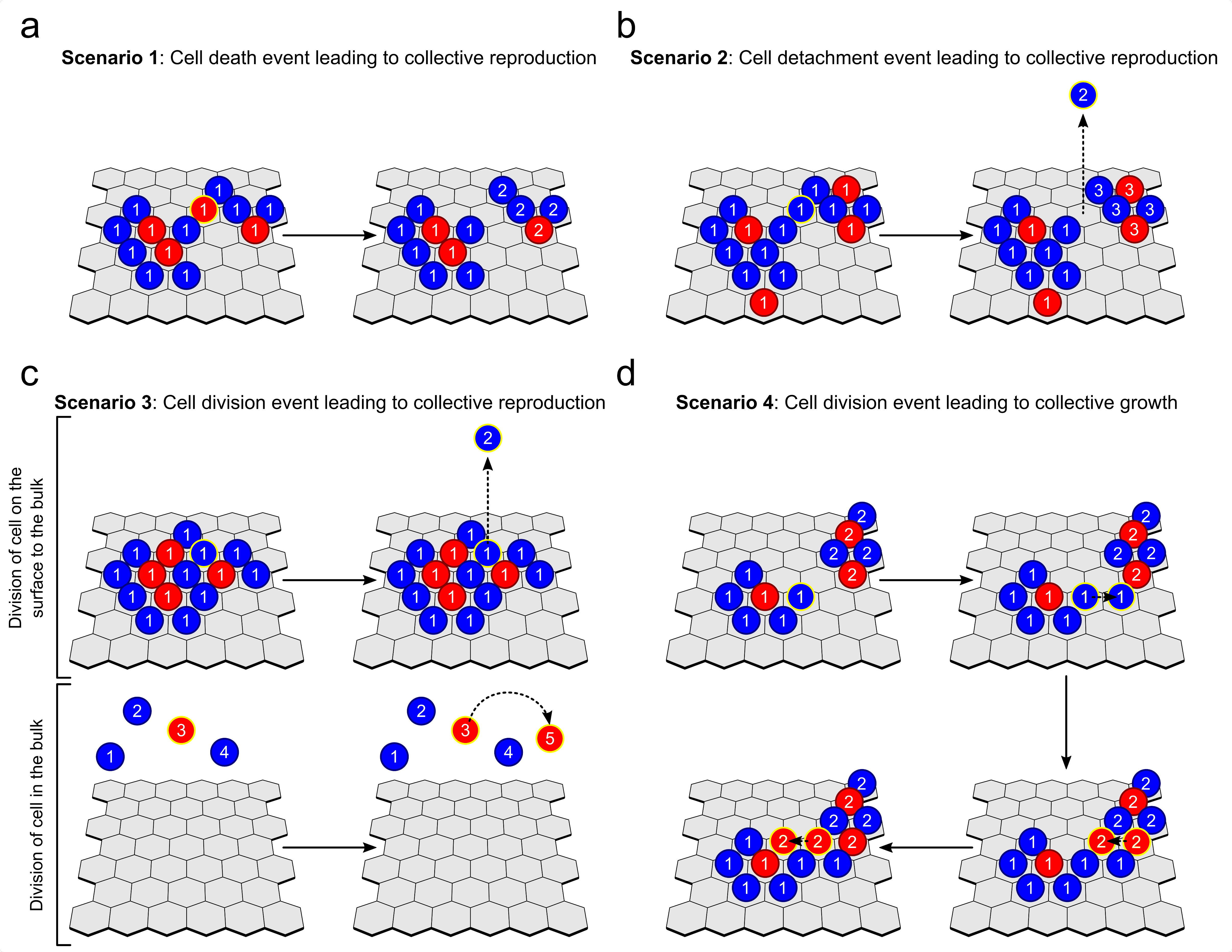

Supplement: S1 Fig — (a) Scenario 1: cell death leading to collective reproduction through fragmentation. Cell death (yellow) can lead to the fragmentation of 2 groups of previously connected cells. (b) Scenario 2: cell detachment leading to reproduction. The detaching cell forms a propagule that originates from within a collective. Detachment can also lead to fragmentation of 2 groups of previously connected cells. (c) Scenario 3: cell division leading to collective reproduction. On a surface, cell division can lead to propagule production when a daughter cell does not remain attached. In the bulk, cells are never associated, so cell division by definition results in reproduction. Note that in the bulk, cells are counted as single cell “collectives.” (d) Scenario 4: cell division leading to collective growth when a daughter cell remains attached to the surface. (TIF) [file pbio.3001250.s001.tif]

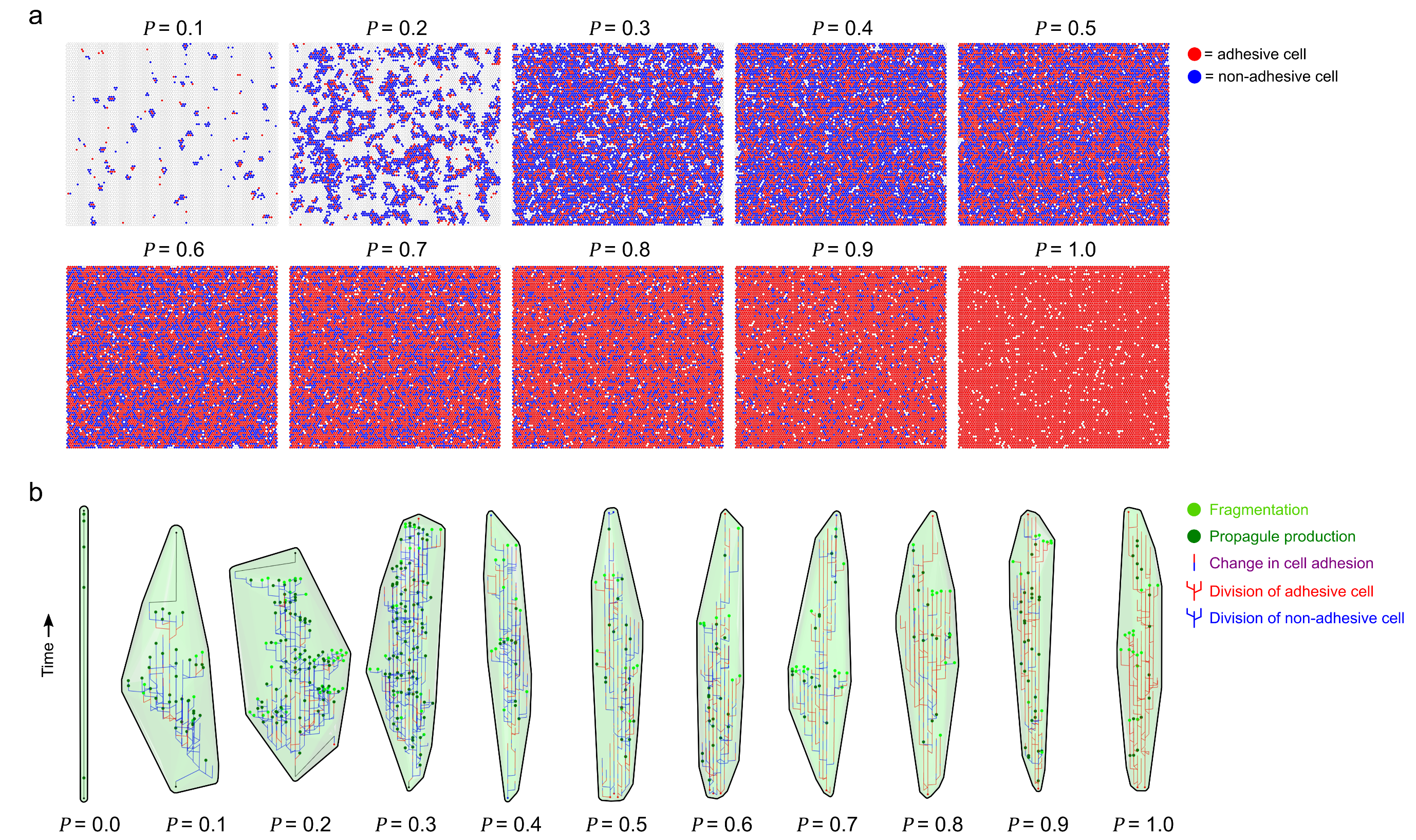

Supplement: S2 Fig — (a) Surface at end of simulations with adhesive (red) and nonadhesive (blue) cells at P = [0.1,1]. (b) Representative cell collectives traced from birth till death (see also Fig 1d): lines, cell lineage underlying cell collective showing both adhesive (red) and nonadhesive (blue) cells; branches, cell division; light green dots, collective offspring resulting from fragmentation; dark green dots, collective offspring resulting from propagule production; green convex hull illustrates relative size of collective. Offspring are not traced in time. Cell lineages show relative location of cells within the collective. (TIF) [file pbio.3001250.s002.tif]

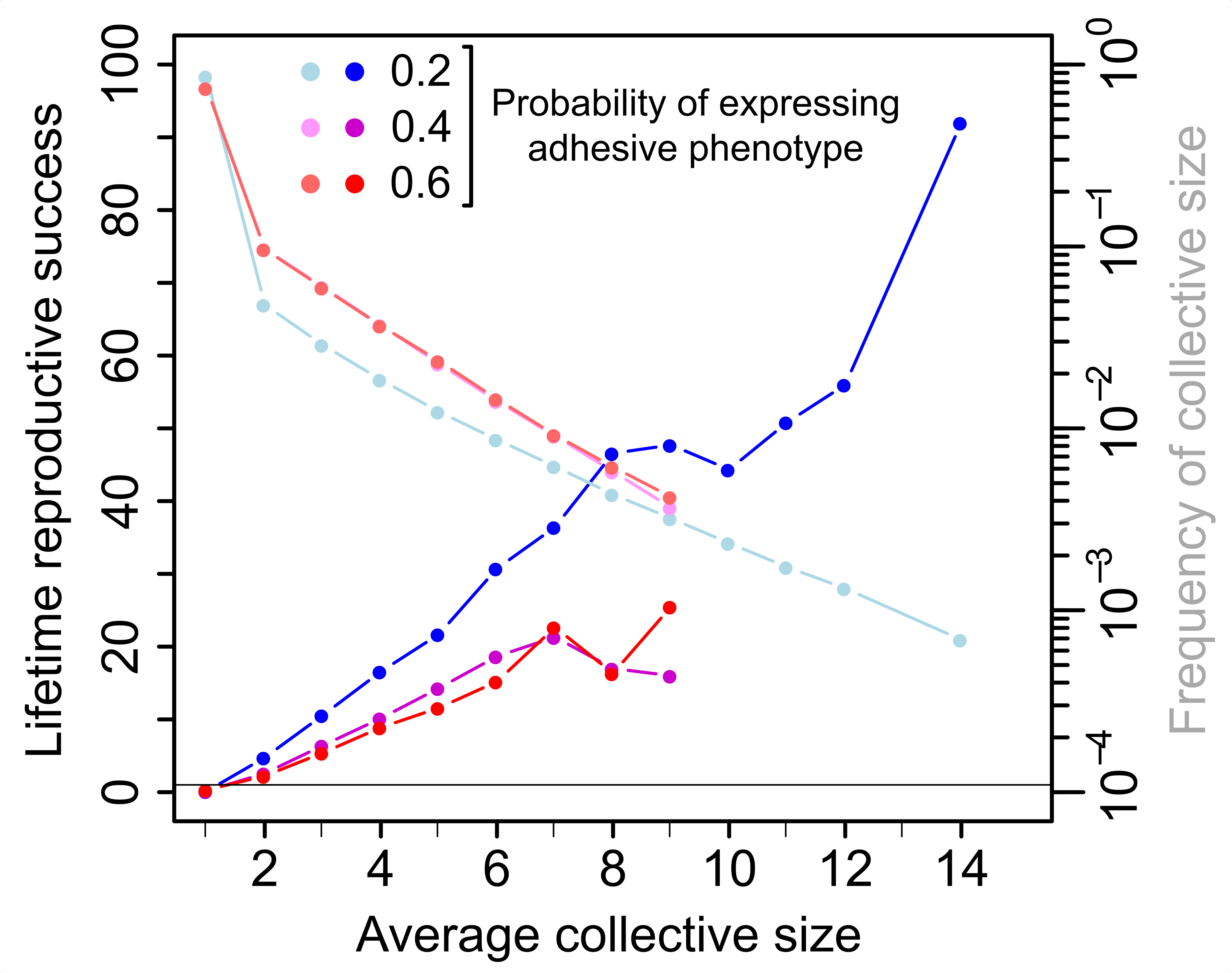

Supplement: S3 Fig — Lifetime reproductive success (left y axis) is determined by the total number of offspring resulting from either propagule production or fragmentation (Fig 1c), produced over the life span of a collective. Average collective size (x axis) refers to the average number of cells that constitute a collective over the life span of the collective. Frequency of collectives (right y axis) with given size. Blue, purple, and red, probability (P) that cells express adhesion of respectively 0.2, 0.4, and 0.6. Raw data are provided in S2 Data in Github repository https://github.com/jordivangestel/PLoS-Biology-2021. (TIF) [file pbio.3001250.s003.tif]

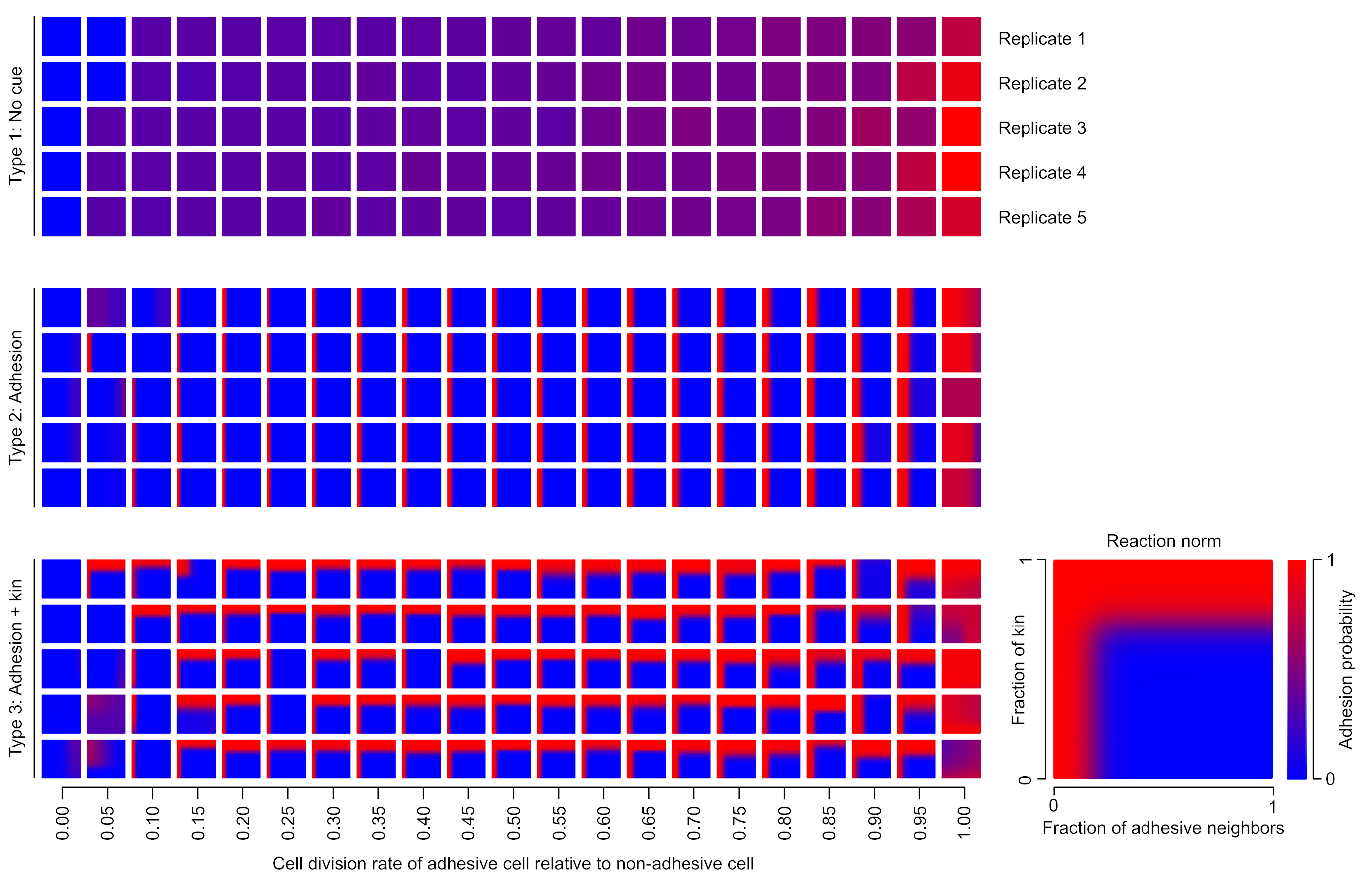

Supplement: S4 Fig — Reaction norms (n = 5) of most abundant genotypes at the end of evolution for different costs of adhesion, where the cost of adhesion is defined by the cell division rate of adhesive cells relative to that of nonadhesive cells. Each reaction norm shows the probability of adhesion (P) as a function of both the fraction of adhesive cells and the fraction of kin in a cell’s neighborhood (i.e., the 6 neighboring positions surrounding a cell on the hexagonal surface). Irrespective of the type of regulation, when the costs of adhesion increase (i.e., lower cell division rate of adhesive cells), the probability of adhesion decreases (i.e., reaction norms color less red and more blue). Within each type of regulation, the shape of the reaction norm remains qualitatively the same for a wide range of adhesion costs. Top-bottom, reaction norms associated with type 1, 2, and 3 regulation. (TIF) [file pbio.3001250.s004.tif]

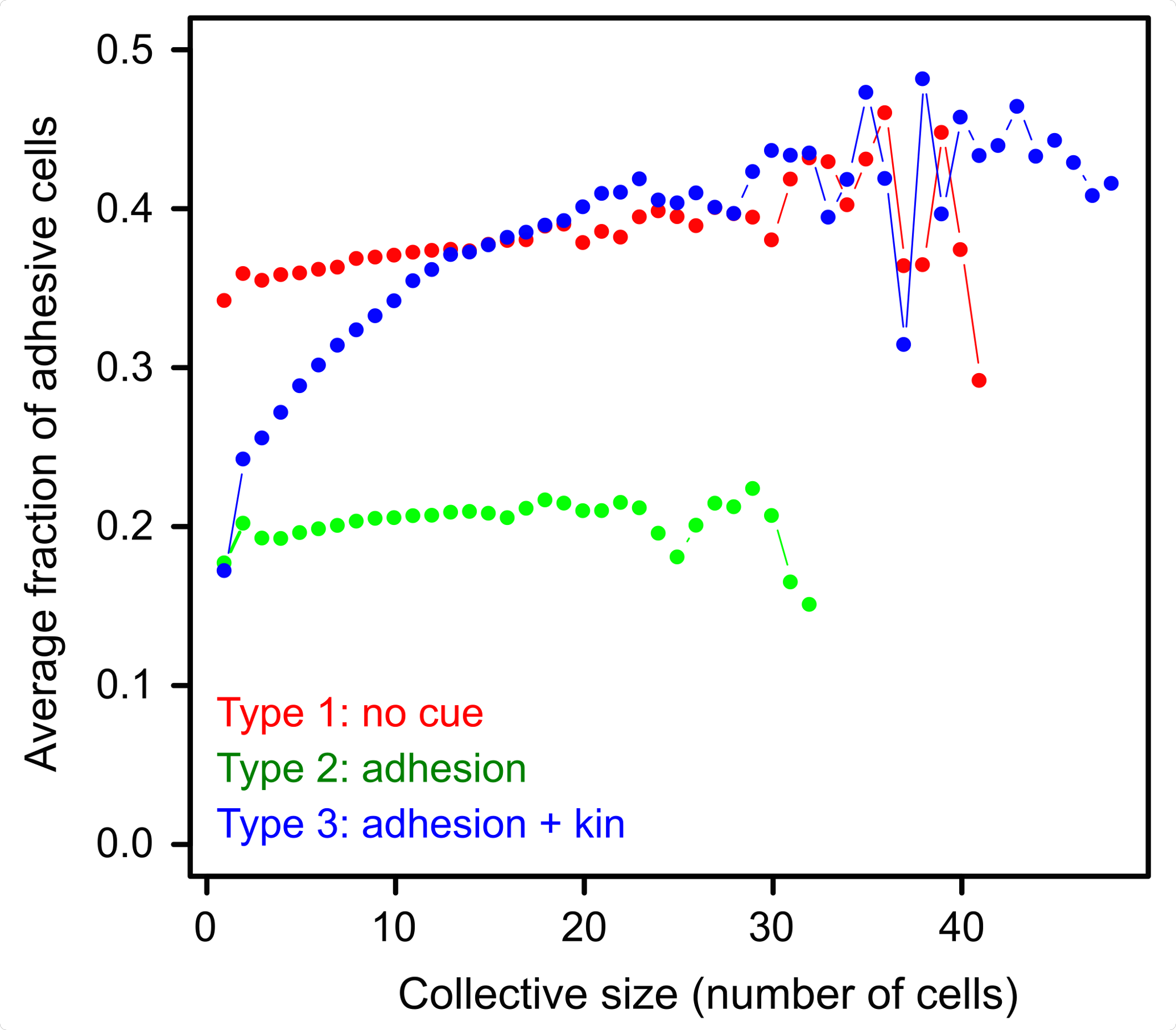

Supplement: S5 Fig — Average fraction of adhesive cells in collectives of different size for type 1 (red), 2 (green), and 3 (blue) regulation. Only in type 3 regulation there is a strong increase in the fraction of adhesive cells when collectives increase in size, while there is only a weak increase for the other types of regulation. Raw data are provided in S2 Data in Github repository https://github.com/jordivangestel/PLoS-Biology-2021. (TIF) [file pbio.3001250.s005.tif]

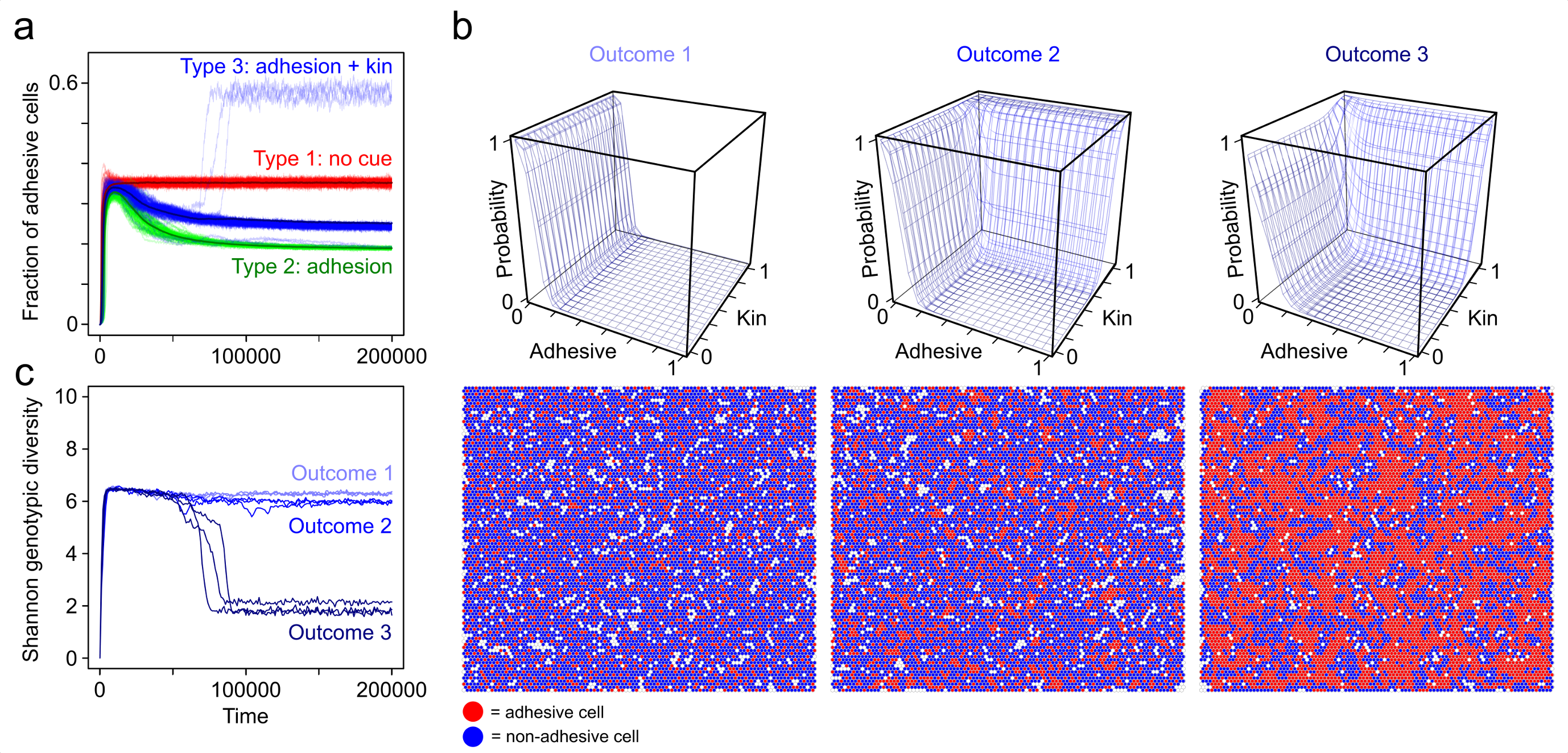

Supplement: S6 Fig — (a) Average fraction of adhesive cells in 100 independent simulations of different type of regulation: red, no cue; green, adhesion only regulation; red, adhesion regulation + kin recognition. (b and c) Detailed analysis of simulations in type 3 regulation. In type 3 simulations, we observe 3 alternative evolutionary outcomes, where fraction of adhesive cells on the surface is (outcome 1, light blue) low, (outcome 2, blue) intermediate, or (outcome 3, dark blue) high. In outcome 1 (3/100 simulations; light blue), kin recognition does not evolve; cells therefore behave the same as those in adhesion only regulation (type 2), which results in a low fraction of adhesive cells on the surface (b). In outcome 2 (94/100, blue), kin recognition evolves, and cells behave as described in the main text (Fig 3), resulting in an intermediate fraction of adhesive cells (b). In outcome 3 (3/100), kin recognition evolves and leads to the dominance of one genotype on the surface. This results in a high fraction of adhesive cells because most cells are surrounded by kin (b). Since one genotype monopolizes the surface, it also results in a collapse of the genotypic diversity in the population, as shown by a strong reduction in the Shannon diversity shown in panel c. (TIF) [file pbio.3001250.s006.tif]

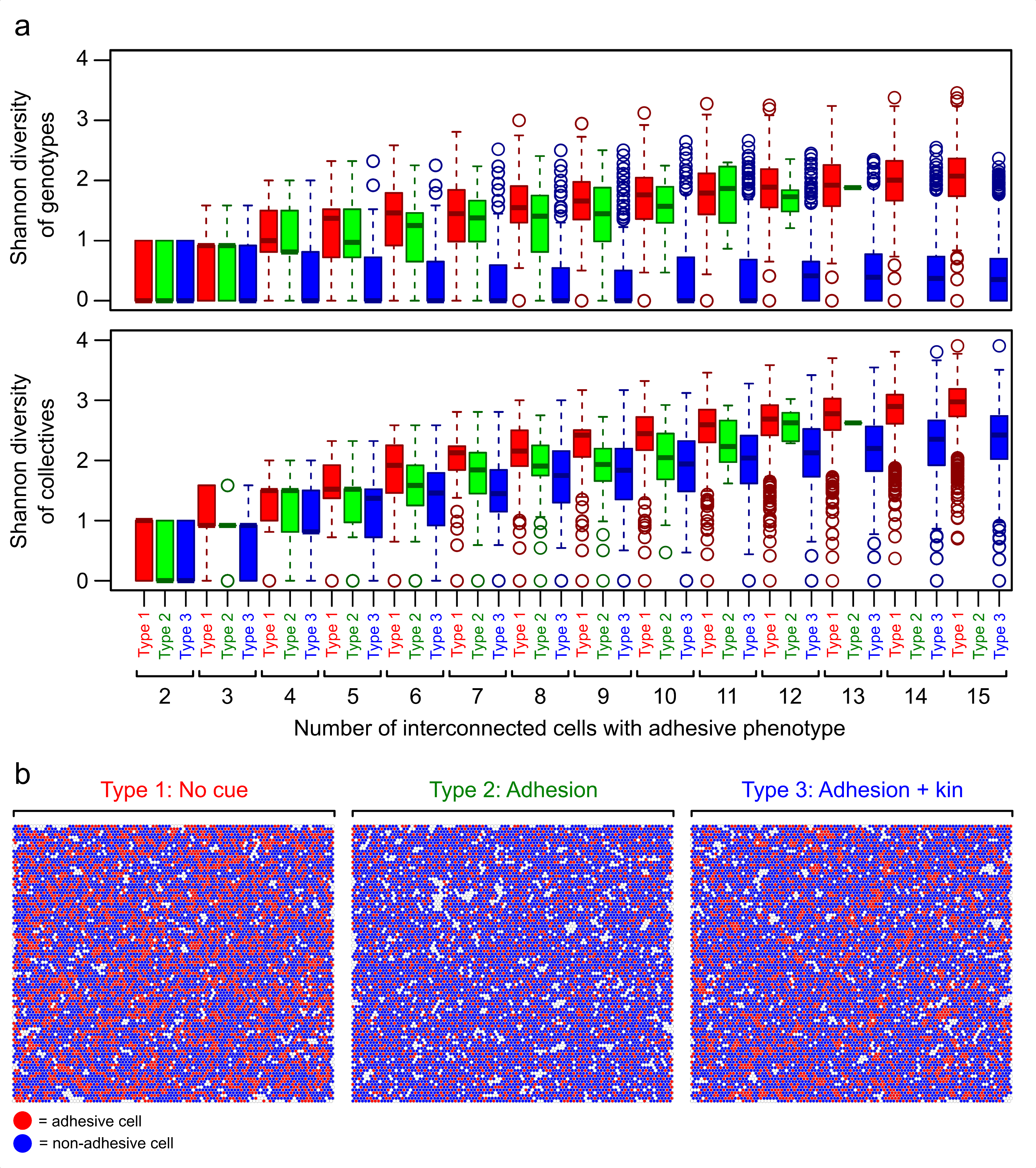

Supplement: S7 Fig — (a) Examination of the diversity underlying clusters of interconnected adhesive cells (directly neighboring each other) on the surface at the end of evolution. For each cluster, we analyze cells one by one and determine their genotype and the collectives to which they belong. Based on this, we calculate the underlying Shannon diversity (H = −∑pi ⋅ ln pi). When all cells in the cluster have the same genotype, the Shannon diversity of genotypes is 0. By the same token, when all cells in the cluster belong to the same collective, the Shannon diversity of collectives is 0. When the Shannon diversity of collectives is higher than 0, it means that the clusters of interconnected cells consists of multiple collectives that merged on the surface. Boxplots in (a) show median Shannon diversity (horizontal black line), interquartile range (box), data within 1.5 times interquartile range above the upper quartile and below the lower quartile (whiskers) and outliers. The diversity analysis uncovers two main findings: First, clusters of interconnected adhesive cells are most homogeneous in type 3 regulation, meaning that they more often consist of cells with the same genotype and cells belonging to the same collective. Thus, in the presence of kin recognition, adhesive cells are more likely to be surrounded by kin than in the absence of kin recognition. Second, for all types of regulation and clusters sizes, the diversity of genotypes is lower than the diversity of collectives, meaning that clusters often consist of multiple collectives that merged on the surface but have the same genotype (hence increasing the diversity of collectives underlying a cluster but not the diversity of genotypes). (b) Surface at the end of evolution for different types of regulation, revealing clusters of interconnected adhesive cells on the surface (i.e., clusters of red cells). Raw data are provided in S2 Data in Github repository https://github.com/jordivangestel/PLoS-Biology-2021. (TIF) [file pbio.3001250.s007.tif]

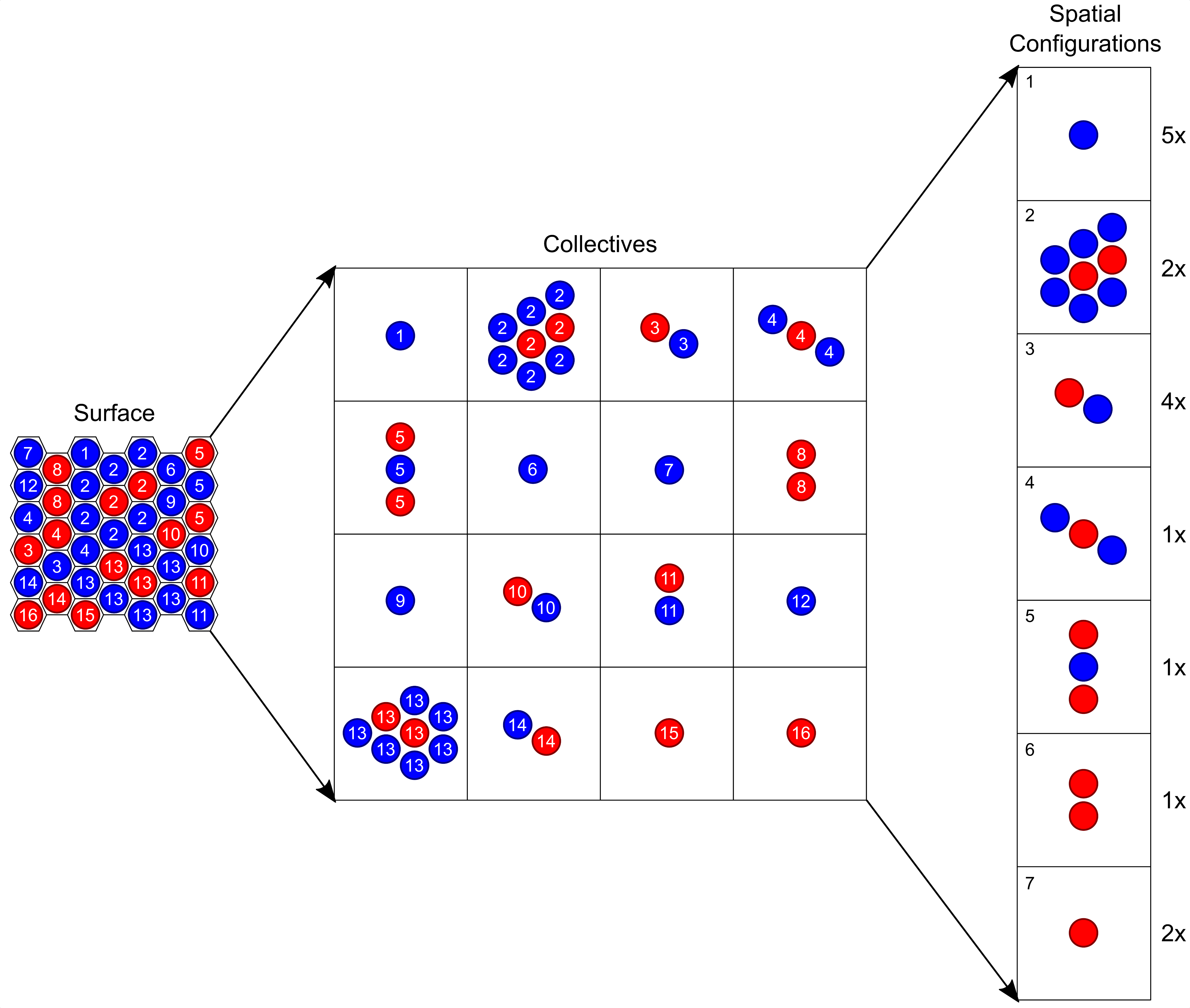

Supplement: S8 Fig — First, all collectives are extracted from the surface, as indicated by the different numbers. Second, the spatial configuration of each collective is determined by examining the spatial location of both adhesive (red) and nonadhesive cells (blue) within the collective. Third, the spatial configurations are compared between collectives to identify all unique configurations, thereby correcting for radial symmetry. This results in a list of unique spatial configurations together with the number of collectives that display each spatial configuration. (TIF) [file pbio.3001250.s008.tif]

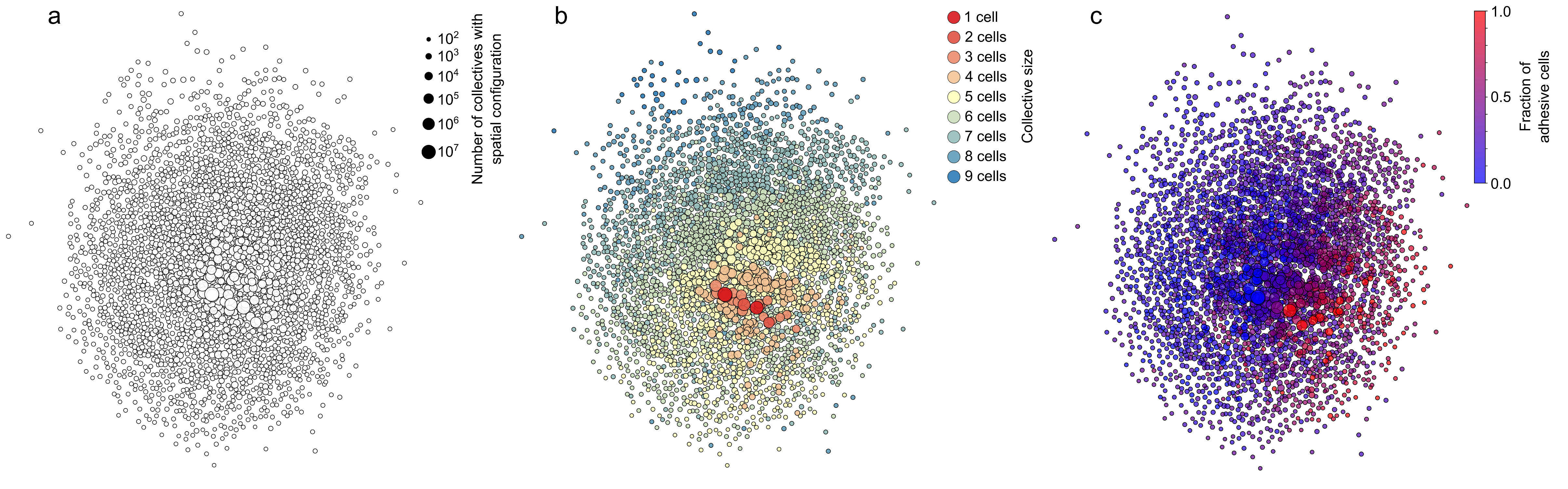

Supplement: S9 Fig — (a) Each node corresponds to a unique spatial configuration that is expressed by at least 100 of 5 ⋅ 107 analyzed collectives. The distance between two nodes is determined by their similarity (see Methods). The node size corresponds to the number of collectives that express the associated spatial configuration. (b) Collective size of different spatial configurations. (c) Fraction of adhesive cells in spatial configuration. (TIF) [file pbio.3001250.s009.tif]

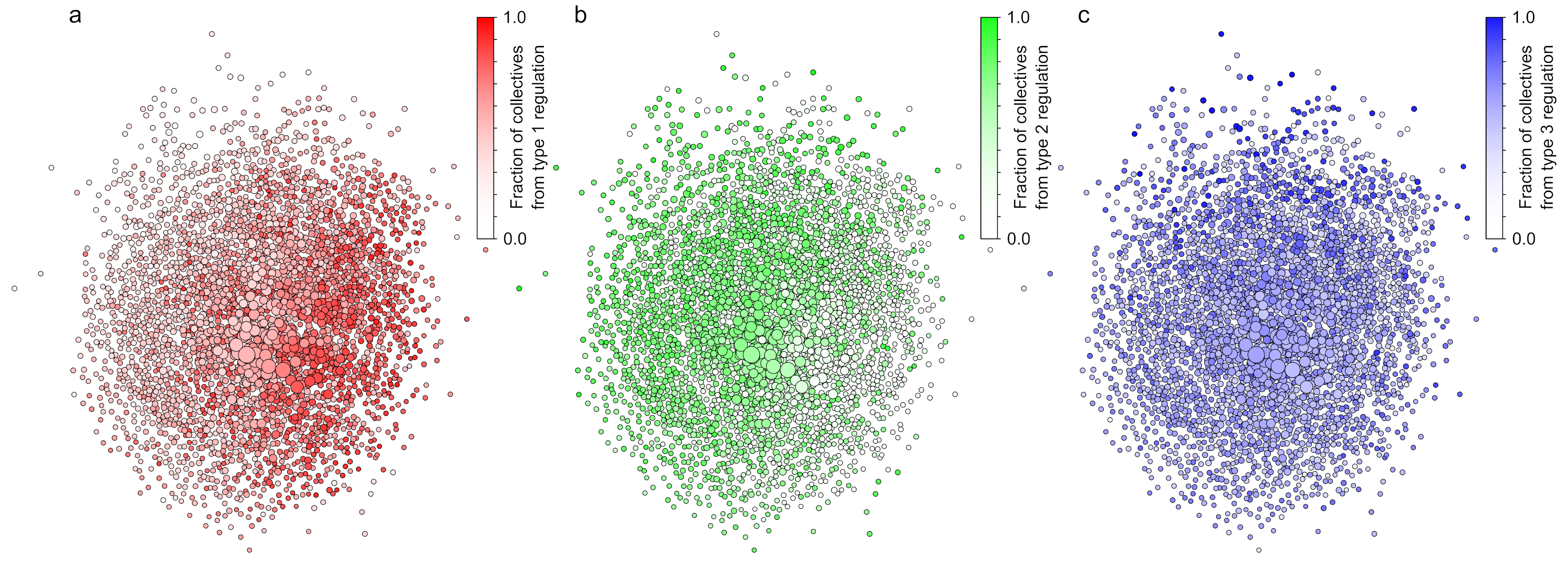

Supplement: S10 Fig — For each spatial configuration (i.e., node in the network), we determine how frequently it is observed among collectives from each type of regulation. This reveals that small collectives with many adhesive cells are more commonly observed in type 1 regulation (a, red), collectives with low fraction of adhesive cells are more commonly observed in type 2 regulation (b, green) and large collectives are more commonly observed in type 3 regulation (c, blue). (TIF) [file pbio.3001250.s010.tif]

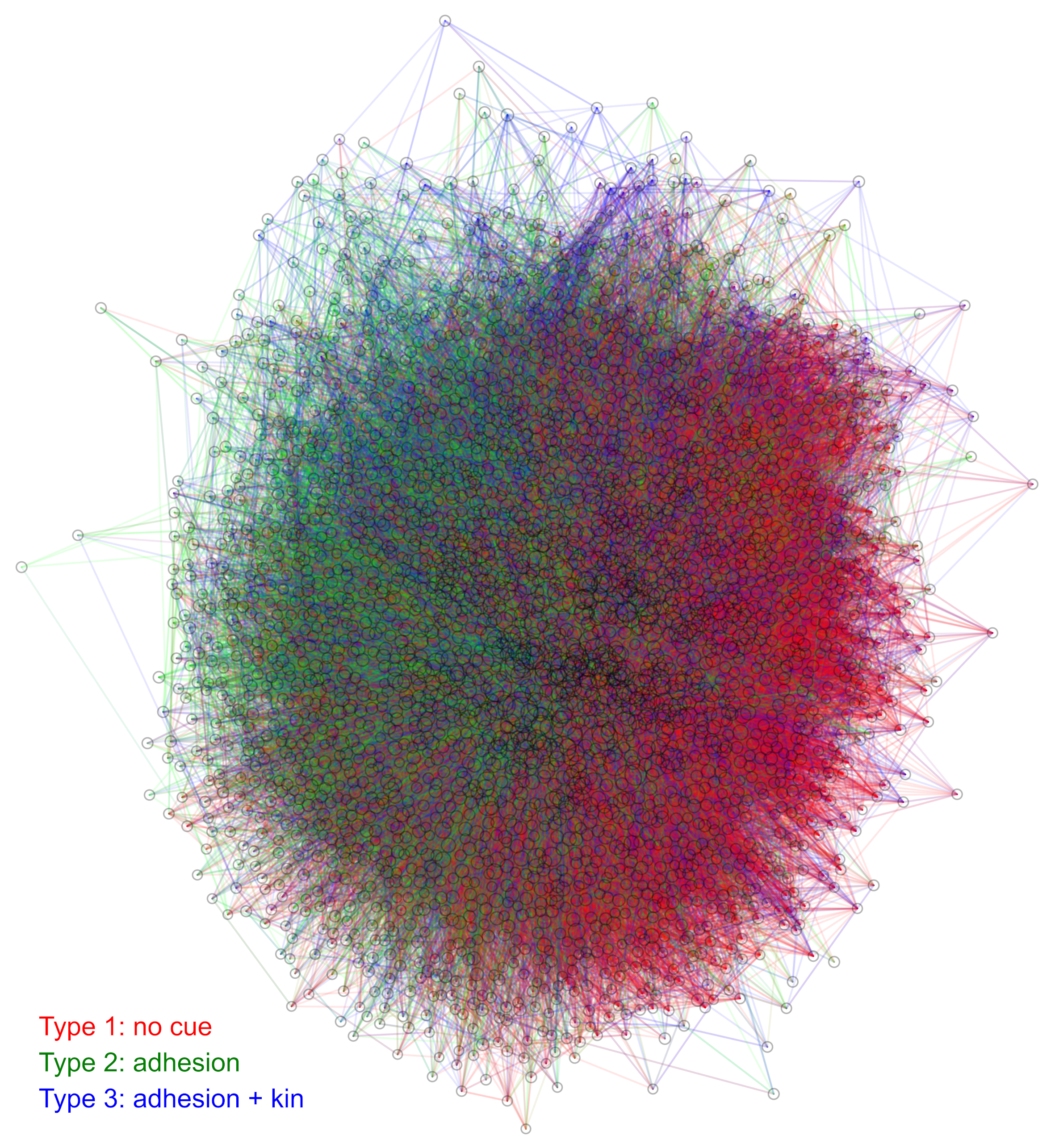

Supplement: S11 Fig — Transparent nodes show unique spatial configurations, like shown in S9 and S10 Figs. Connections show how collectives change their spatial configuration in time (see also S12 Fig for individual examples). Color shows frequency by which change in spatial configuration is observed in different types of regulation: red, changes mostly observed in type 1 regulation; green, changes mostly observed in type 2 regulation; blue, changes mostly observed in type 3 regulation. (TIF) [file pbio.3001250.s011.tif]

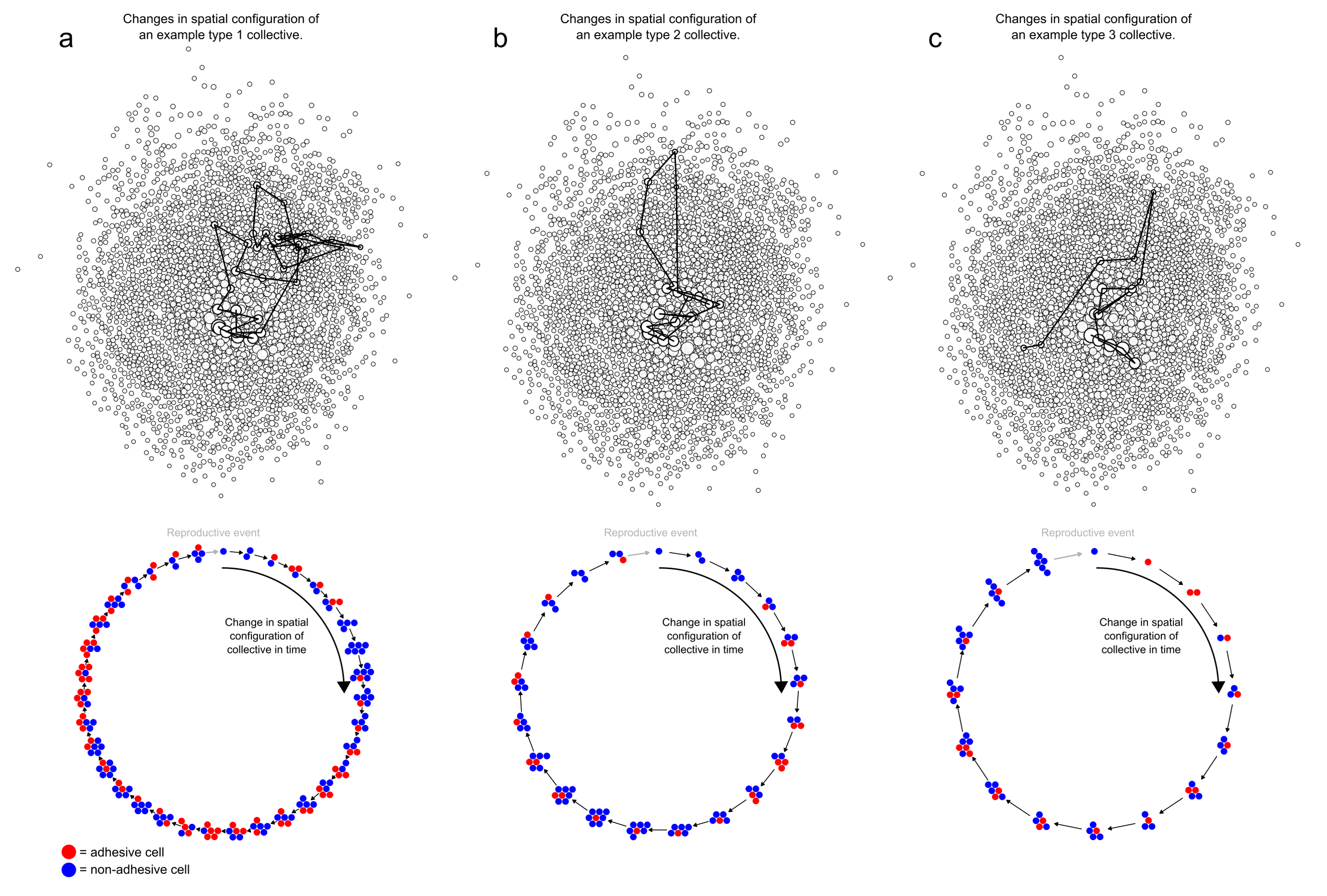

Supplement: S12 Fig — Each panel shows an example of how a collective in one of the 3 regulatory systems changes its spatial configuration in time. In each example, the collective is seeded by a nonadhesive cell, which grows on the surface and thereby gives rise to different spatial configurations. Upper, bold black lines show within the network representation how a collective changes its spatial configuration in time. Below, small representations of collective show how the spatial configuration of the collective changes in time. We trace each collective until the first successful reproductive event that leads to a nonadhesive propagule (small grey arrow), thereby completing the life cycle. (a) Example of how collective changes its spatial configuration in the type 1 regulation. (b) Example of how collective changes its spatial configuration in the type 2 regulation. (c) Example of how collective changes its spatial configuration in the type 3 regulation. (TIF) [file pbio.3001250.s012.tif]

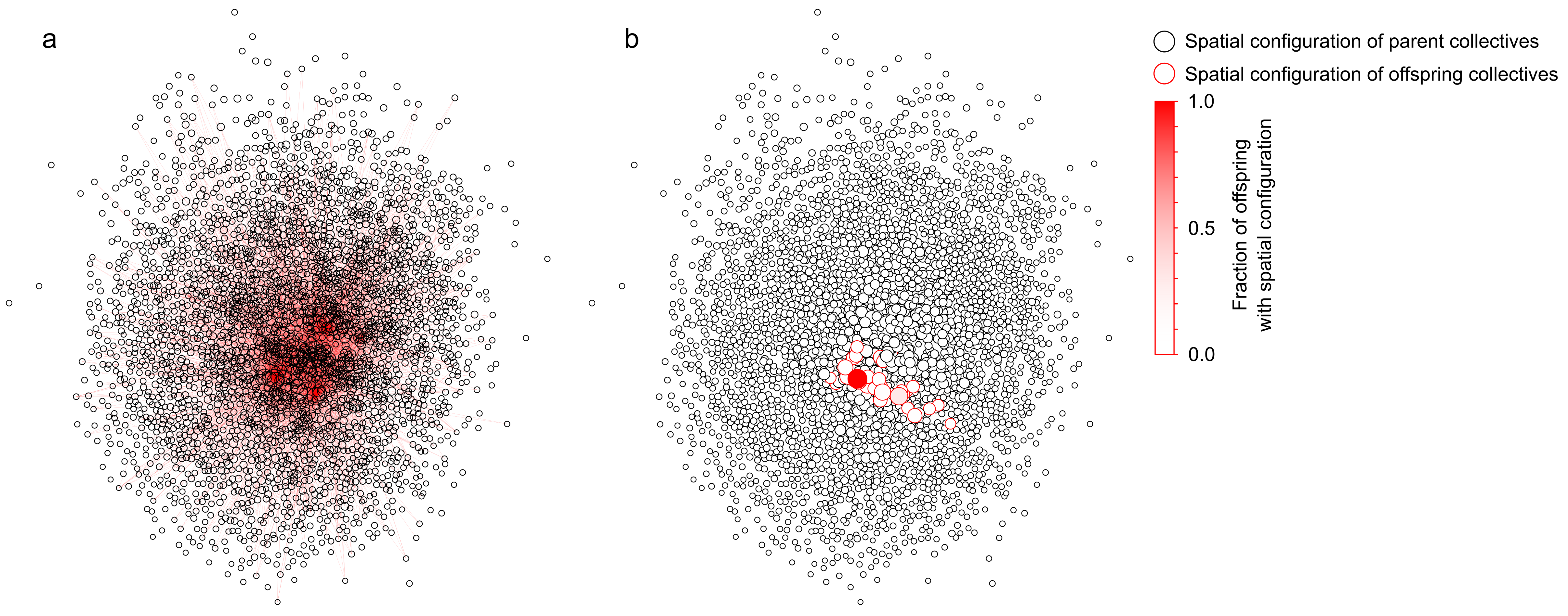

Supplement: S13 Fig — (a) Lines connect spatial configuration of parental collectives and offspring collectives upon reproduction. (b) Red nodes highlight spatial configuration of offspring collectives. The frequency by which a configuration is observed is indicated by the saturation of the fill color. Most offspring collectives have the same spatial configuration: nonadhesive single-cell propagules (see also S9 Fig). (TIF) [file pbio.3001250.s013.tif]
