# Supplementary material for: Cryptic surface-associated multicellularity emerges through cell adhesion and its regulation
Source: PLoS Biol. 2021 May 13;19(5):e3001250. doi: 10.1371/journal.pbio.3001250 (PMC8148357; doi:10.1371/journal.pbio.3001250)
Supplement: S1 Text — Description of model, which includes a description of all cell level events, determination of the adhesion probability for the 3 different types of regulation, pseudocode, simulation conditions, and a discussion on the role of the surface geometry. (PDF) [file pbio.3001250.s027.pdf]

S1 Text. Model

Cell level events

In the model, we explicitly account for cell-level events only. Cells can express two discrete phenotypes, adhesive or not. Adhesion is required for surface attachment. We assume that adhesion mediates both cell-surface contact and cell-cell contact on a surface. Accordingly, cells can attach to a surface when they are adhesive themselves or associate with an adhesive cell on a surface. Given the hexagonal geometry of the surface, an adhesive cell can support the attachment of up to six non-adhesive neighbors. In the bulk, cell do not adhere to each other. We assume time is discrete. At each time step, we randomly sample the population cell by cell, each of which can undergo one of the five possible cell-level events: (i) surface attachment, (ii) surface detachment, (iii) cell division, (iv) cell death or (v) changes in cell adhesion. Here, we detail each of these events:

1. **Surface attachment**. Cells from the bulk can attach to the surface with a probability of 10% ($P_a = 0.1$). In the case of attachment, cells associate with a random grid element on the surface. Attachment is successful when there is no other cell at this grid element and when the cell or one of its neighbors is adhesive. Thus, when the surface occupancy increases, it becomes more difficult for a cell to attach to the surface (i.e. fewer available grid elements).
2. **Surface detachment**. Cells detach from the surface whenever they and their neighbors lack adhesion. By detaching, cell moves to the bulk, which frees up the grid element on the surface and increases the population size in the bulk by one cell. Detachment occurs irrespective of the population size in the bulk and could lead to overshooting the carrying capacity ($K_{bulk} = 5.000$ cells). When the population size exceeds the carrying capacity at the end of a timestep, we randomly remove cells from the bulk till the population size equals the carrying capacity, thereby mimicking a chemostat model where cells are diluted out of the population.
3. **Cell division**. Cell division depends both on a cell's phenotype and environment. We assume adhesion is costly (C_a) and lowers the division rate of adhesive cells with 60% compared to non-adhesive cells. We purposely implement such high costs to examine a worst-case scenario, but similar results are obtained for a wide range of adhesion costs (S4 Fig). Cell division differs for cells in the bulk and on the surface. In the bulk, we assume strong competition and cells can only divide when the population size is below the carrying capacity ($K_{bulk} = 5.000$ cells). On the surface, a cell can divide whenever there is space in one of the neighboring grid elements. A daughter cell only remains attached when it is adhesive or surrounded by an adhesive neighbor. In lack of space, daughter cells can also

immediately detach to the bulk, but we assume this is only possible when the population size in the bulk is below the carrying capacity. With this implementation, cells on the surface only have an enhanced division probability compared to cells in the bulk when there is space. Without surface space, cells in the bulk and on the surface have the exact same division probability (when they have the same phenotype). Both on the surface and in the bulk, we assume that daughter cells adopt the same phenotype as their mother upon division. In the evolutionary simulations (Fig 2f, 3b and S4, S6 Fig), we assume that daughter cells have a small probability of incurring a mutation that affects the adhesion probability, as described below.

4. **Cell death**. Cells die with a fixed probability of 10% ($P_d = 0.1$) irrespective of the environment and phenotype of a cell.
5. **Changes in cell adhesion**. Cells become (or remain) adhesive with probability, P, and non-adhesive with probability, $1 - P$. Depending on the type of regulation (see Fig 3 and below), the adhesion probability can depend on the fraction of adhesive neighbors and kin, which we discuss in the next section.

Adhesion probability

In our model, we implement different types of regulation underlying the adhesion probability. The types differ in the amount of information cells can obtain from their neighbors. In the first type, cells have no information about their neighbors, in the second type, cells sense the fraction of adhesive neighbors and, in the third type, cells – in addition to adhesion – sense the fraction of neighbors from the same kin group (assuming the presence of a kin recognition system). We purposely consider these nested types of regulation because it allows us to determine how every additional cue affects the evolution of collectives. Here, we describe the three types of regulation in detail:

- **Type 1.** In this type of regulation, cells have no information about their environment and become adhesive with a fixed probability, P. In evolutionary simulations (Fig 2f and 3), this probability can mutate ($\mu_r = 5 \cdot 10^{-3}$). When a mutation occurs, a value is added to P that is drawn from a normal distribution $N(0, \mu_s)$ (for Fig 2f we assume that $\mu_s = 0.02$ and for Fig 3 we assume that $\mu_s = 0.1$). P is constraint between $[0\ 1]$.
- **Type 2**. In the second type, cells sense the fraction of adhesive neighbors (f_a). The adhesion probability (P) depends on f_a according to the following sigmoidal reaction norm:

$$[1]\quad P(f_a) = P_{min} + \frac{P_{max} - P_{min}}{1 + e^{a_1 \cdot F \cdot (f_a - b_1)}}$$

The adhesion probability $P(f_a)$ ranges from a minimal probability of P_{min} to a maximum adhesion probability of P_{max}. Within this range, the adhesion probability can either increase ($a_1 < 0$) or decrease ($a_1 > 0$) with the fraction of adhesive cells (f_a), following a sigmoidal curve. When $a_1 = 0$, the fraction of adhesive cells has no effect on the adhesion probability. The inflection point of the sigmoidal curve is determine by b_1. F is a scaling parameter, which is fixed, and magnifies the effect of mutational changes in a_1 to facilitate evolution ($F = 20$). At the onset of evolution (Fig 3), cells are not adhesive and do not sense the fraction of adhesive neighbors ($P_{min} = P_{max} = a_1 = 0$ and $b_1 = 0.5$). Then, at every cell division, the genotypic parameters $[P_{min}, P_{max}, a_1, b_1]$ can mutate at a rate $\mu_r = 5 \cdot 10^{-3}$. When a mutation occurs, a value is added to the genotypic parameter that is drawn from a normal distribution $N(0, \mu_s)$. P_{min} and P_{max} are constraint between [0 1], such that $0 \leq P \leq 1$.

- Type 3. In the third type of regulation, cells can sense both the fraction of adhesive cells (f_a) and the fraction of cells from the same kin group (f_k). A kin group is here defined by the genotypic parameters, such that cells from the same kin group respond identically to their neighbors. The adhesion probability (P) depends on f_a and f_k according to the following two-dimensional sigmoidal reaction norm:

$$[2]\quad P(f_a, f_k) = P_{min} + \frac{P_{max} - P_{min}}{(1 + e^{a_1 \cdot F \cdot (f_a - b_1)}) \cdot (1 + e^{a_2 \cdot F \cdot (f_k - b_2)})}$$

The adhesion probability $P(f_a, f_k)$ ranges from a minimal probability of P_{min} to a maximum adhesion probability of P_{max}. Within this range, the adhesion probability can either increase ($a_i < 0$) or decrease ($a_i > 0$) with fraction of adhesive cells (f_a) and kin (f_k), following a two-dimensional sigmoidal curve. When $a_i = 0$, a cue has no effect on the adhesion probability. The inflection points are determined by b_1 and b_2. F is a scaling parameter, which is fixed, and magnifies the effect of mutational changes in a_i to facilitate evolution ($F = 20$). At the onset of evolution (Fig 3), cells are not adhesive and do not sense their neighbors: $P_{min} = P_{max} = a_1 = a_1 = 0$ and $b_1 = b_2 = 0.5$. Then, at every cell division, each genotypic parameter can mutate at a rate $\mu_r = 5 \cdot 10^{-3}$. When a mutation occurs, a value is added to the genotypic parameter that is drawn from a normal

distribution $N(0, \mu_s)$. The parameter values of P_{min} and P_{max} are constraint between $[0\ 1]$, such that $0 \leq P \leq 1$.

Pseudocode

```
Parameters
T_max       = 200000    // Duration of simulation in number of timesteps

K_bulk      = 5000      // Carrying capacity of population in bulk
K_surface   = 10000     // Carrying capacity on surface, dimension: 100x100

P_a         = 0.1       // Attachment probability
P_d         = 0.1       // Death rate
C_a         = 0.6       // Reduced cell division rate of adhesive cells
mu_r        = 5*10^-3   // Mutation rate per division per genotypic parameter
mu_s        = 0.1       // Mutation size

Struct Cell
{
      P           // Probability of adhesion
      Location    // Location: either bulk or surface
      Phenotype   // Either adhesive or not
      Genotype    // Parameters describing reaction norm
      // Genotype for type 1 regulation: [P]
      // Genotype for type 2 regulation: [P_min, P_max, a_1, b_1]
      // Genotype for type 3 regulation: [P_min, P_max, a_1, b_1, a_2, b_2]
}

State variables
vector<Cell> Population             // Population of cells
vector<int> Bulk                    // Keeps track of cells in bulk
vector<vector<int>> Surface         // Keeps track of cells on grid

Functions
attachment(ind) // Event 1
{
      if random_uniform(0,1) < P_a do     // When random number is below P_a
      {
            if ind.location == Surface do nothing
            if ind.location == Bulk do
            {
                  1. Pick random location on grid
                  2. Determine if location is available
                  3. Determine if cell is adhesive or has adhesive neighbors
                  4. If (2) and (3) are true, attach cell to grid
            }
      }
}
detachment(ind) // Event 2
{
```

```
        if ind.location == Bulk do nothing
        if ind.location == Surface do
        {
              1. Is cell non-adhesive (ind.phenotype = non-adhesive)
              2. Are neighbors non-adhesive
              3. If (1) and (2) are true, detach cell to bulk
        }
}
division(ind) // Event 3
{
        if ind.phenotype == adhesive do P_division = 1- C_a   // Adhesion costs
        if ind.phenotype == non-adhesive do P_division = 1
        if random_uniform(0,1) < P_division do
        {
              if ind.location == Bulk do
              {
                    1. Check if population size in bulk < K_bulk
                    2. If (1) is true, divide
                    3. In evolutionary simulations, possibility of mutation
              }
              if ind.location == Surface do
              {
                    1. Check if there is space on surface or in bulk.
                          a. Surface: Check neighboring grid elements that are
                             empty. Ignore grid elements where daughter cell
                             cannot remain attached due to lack of adhesion.
                          b. Bulk: Check if population size is below K_bulk
                    2. If there is space, divide. Daughter cell randomly goes
                       to either one of the neighboring grid elements with
                       adhesion or to the bulk.
                    3. In evolutionary simulations, possibility of mutation
              }
        }
}
death(ind) // Event 4
{
        if random_uniform(0,1) < P_death do remove cell from population
}
adhesion(ind) // Event 5
{
        ind.P = function()            // For type 1 regulation
        ind.P = function(f_a)         // For type 2 regulation
        ind.P = function(f_a,f_k)     // For type 3 regulation
        if random_uniform(0,1) < ind.P do
        {
              ind.phenotype = adhesive
        }else{
              ind.phenotype = non-adhesive
        }
}
//Main loop
for t from 1 to T_max do
{
```

```
        Population = shuffle(Population)    // Shuffle vector of cells
        for i from 1 to size(Population) do
        {
              Ind = Population[i]           // Take individual cell
              Event = random_integer(1,5)   // Determine random event 1-5
              if Event == 1, do attachment(Ind)
              if Event == 2, do detachment(Ind)
              if Event == 3, do division(Ind)
              if Event == 4, do death(Ind)
              if Event == 5, do adhesion(Ind)
        }
}
```

Simulations

We run two different sets of simulations: non-evolutionary simulations, where we explore how cells with a fixed adhesion probability self-organize on the surface and give rise to collectives (Fig 2a-e), and evolutionary simulations, where we explore how the adhesion probability evolves and thereby affects the emergent collectives (Fig 2f and 3). In both evolutionary and non-evolutionary simulations, we start with a population of non-adhesive cells in the bulk at carrying capacity ($K_{bulk} = 5.000$ cells), which can subsequently become adhesive and colonize the surface. In the non-evolutionary simulations (Fig 3a-c, f), we run the simulations for 1000 timesteps ($T_{max} = 1000$), which equals approximately 20 cell generations, and use the last 200 timesteps for spatial lineage tracking of cells ($T_{track} = 200$). As detailed in S2 Text, spatial lineage tracking is used to study the cell collectives that spontaneously emerge on the surface. We only track cells at the end of the simulations, because the population size needs time to equilibrate (when cells first colonize the surface, the population size increases). For Fig 2d (and S2b Fig), we run the simulations slightly longer ($T_{max} = 2000$, $T_{track} = 500$) to include more collectives in our analysis. Note that a small fraction ($\ll 0.1\%$) of collectives has a lifespan that is larger than 500 timesteps. Since we only track cells for 500 timesteps, these collectives are not included in the analysis of Fig 2d (and S3 Fig), where we quantify lifetime reproductive success. Unless otherwise noted, we ran 10 replicate simulations for each parameter setting.

For the pairwise invasibility plot (Fig 2e), we competed different combination of resident (P_r) and mutant (P_m) genotypes, which express different adhesion probabilities. For each combination, we start with a population of 5000 non-adhesive cells in the bulk, of which 10% (f_{start}) carries the mutant genotype (P_m) and 90% the resident phenotype (P_r). Genotypes are subsequently allowed to compete for 1000 timesteps (T_{max}), after which we measured the fraction of mutant genotypes, f_{end}. We perform 10

replicate simulations for each combination and use the average frequency of the mutant genotype at the end of evolution, $\langle f_{end} \rangle$, to calculate the invasion fitness: $log_2\left(\frac{\langle f_{end} \rangle}{f_{start}}\right)$.

The evolutionary simulations were ran for 200.000 timesteps ($T_{max} = 200.000, T_{track} = 200$), which equals approximately 4000 cell generations. For each type of regulation, we performed 100 replicates (S6 Fig), of which the first 10 are analyzed in detail (Fig 3). For these simulations, we also performed a detailed analysis of how the evolved collectives change in time (S3 Text).

All simulations were performed in C++ using Visual Studio 2012, the cpp source codes are included as S1 Data in Github repository https://github.com/jordivangestel/PLoS-Biology-2021.

The role of surface geometry

We examined surface colonization on a simple two-dimensional hexagonal surface geometry. In nature, surface-colonizing organisms rarely show strict two-dimensional growth. Instead they often accumulate biomass through both lateral and vertical colony expansion [1–3]. In addition, surface colonizers often express distinct cell types that directly impact surface growth [4]. For instance, they can overcome surface tension and thereby facilitate lateral expansion through filamentous growth. The simple surface geometry in our model, where cells are physically identical (i.e., of the same size and shape) and can only proliferate on a two-dimensional plane, purposely sidesteps these biological and biophysical details, in order to illustrate our method of spatial lineage tracking. However, in a previous version of the model [5], which builds on similar modeling premises (but where we did not yet apply our method of spatial lineage tracking to study the early emergence of collectives), we have relaxed these assumptions. For instance, we explored alternative surface geometries, including triangular, squared and hexagonal geometries. These alternative geometries did not qualitatively impact the outcome of the model. This can be explained by the fact that – irrespective of the exact geometry – local interactions between neighboring cells are the same, leading to similar selective pressures on the surface: whether a cell has three (triangular) or six neighbors (hexagonal), it can only remain attached when it is adhesive or if one of its neighbors is adhesive. As a consequence, cells evolve a similar dependency on the adhesive properties of their neighbors [5]. In addition, we previously explored simple three-dimensional implementations of our model [5]. These also had little impact on the modeling outcome. This can be explained by the fact that – within our minimal modeling assumptions – surface growth is radially symmetrical, resulting in similar growth dynamics in all dimensions. In fact, this radial symmetry is also apparent in in our current model, where collectives are best described by their size and central-peripheral polarity in adhesion (Fig 3d,e).

We emphasize that surface growth is not necessarily radially symmetrical in nature, since the biophysical conditions (e.g., adhesive forces, flow) close to the surface can be markedly distinct from those away from the surface. This is illustrated by fruiting bodies and sporangia, which protrude from the surface in order to escape from the adhesive forces that play a dominant role close to the surface. In addition, cells close to the surface may have access to fewer or more resources, depending on whether resources are extracted from the growth substrate or the surroundings. An exciting task for future work is to apply our method of spatial lineage tracking to real-world examples of surface growth, which account for the biophysical and biological details of colony growth, thereby determining how these details affect the emergence and evolution of surface-associated multicellularity. We purposedly sidestepped these details in our current model, in order to illustrate the central principles at work most clearly.

References

1. Warren MR, Sun H, Yan Y, Cremer J, Li B, Hwa T. Spatiotemporal establishment of dense bacterial colonies growing on hard agar. Neher RA, Barkai N, Neher RA, editors. eLife. 2019;8: e41093. doi:10.7554/eLife.41093

2. Qin B, Fei C, Bridges AA, Mashruwala AA, Stone HA, Wingreen NS, et al. Cell position fates and collective fountain flow in bacterial biofilms revealed by light-sheet microscopy. Science. 2020;369: 71–77. doi:10.1126/science.abb8501

3. Drescher K, Dunkel J, Nadell CD, van Teeffelen S, Grnja I, Wingreen NS, et al. Architectural transitions in *Vibrio cholerae* biofilms at single-cell resolution. Proc Natl Acad Sci USA. 2016;113: E2066-2072. doi:10.1073/pnas.1601702113

4. van Gestel J, Vlamakis H, Kolter R. From cell differentiation to cell collectives: *Bacillus subtilis* uses division of labor to migrate. PLoS Biol. 2015;13: e1002141. doi:10.1371/journal.pbio.1002141

5. van Gestel J, Nowak MA. Phenotypic heterogeneity and the evolution of bacterial life cycles. PLoS Comput Biol. 2016;12: e1004764. doi:10.1371/journal.pcbi.1004764
