# Supplementary material for: Cryptic surface-associated multicellularity emerges through cell adhesion and its regulation
Source: PLoS Biol. 2021 May 13;19(5):e3001250. doi: 10.1371/journal.pbio.3001250 (PMC8148357; doi:10.1371/journal.pbio.3001250)
Supplement: S2 Text — Description of collective growth, reproduction, and death. (PDF) [file pbio.3001250.s028.pdf]

S2 Text. Spatial lineage tracking

Since our model only accounts for cell-level events, we need to monitor when and how collectives emerge on the surface. We do this using spatial lineage tracking of cells, where we follow all cells in time. In this section, we briefly explain how cell-level events (surface attachment, surface detachment, cell division, cell death) affect collective growth, reproduction and death.

Collective growth. A surface-associated collective grows when one of its constituent cells divides and the daughter cell remains attached to the surface (S1d Fig). Collective growth always results from cell division, but not every cell division leads to collective growth, because – as describe in S1 Text (see also Fig 2c) – the daughter cell could also immediately detach to the bulk.

Collective reproduction. A surface-associated collective reproduces when cells constituting the collective somehow disassociate from each other. This could either result from fragmentation or propagule production. In the case of fragmentation, a collective on the surface separates into two or more smaller fragments due to the detachment of cell from the surface or cell death (S1a,b Fig). By definition, we consider the largest fragment as the parental collective and the remaining fragment(s) as its offspring. When the fragments are equally big, the parent is assigned randomly. In the case of propagule production, cell detach from the surface and move to the bulk. This could either result from a lack of adhesion (S1b Fig), which inevitably leads to detachment, or from cell division, where the daughter could immediately detach following cell division (S1c Fig). In order to include all cells in our analysis of collectives, we also consider ‘collectives’ that consist of single cells only. Accordingly, in our analysis, all cells in the bulk are counted as single-cell collectives. Thus, when cells in the bulk divide, this – by definition – leads to collective production.

Collective death. A collective dies when the last surviving cell constituting the collective ceases to exist. The death of the collective therefore always coincides with a cell death event.
