# Supplementary material for: Cryptic surface-associated multicellularity emerges through cell adhesion and its regulation
Source: PLoS Biol. 2021 May 13;19(5):e3001250. doi: 10.1371/journal.pbio.3001250 (PMC8148357; doi:10.1371/journal.pbio.3001250)
Supplement: S3 Text — Analysis of growth dynamics of collectives. (PDF) [file pbio.3001250.s029.pdf]

S3 Text. Growth dynamics of collectives

Besides analyzing the static properties of collectives at the end of evolution, as shown in Fig 3, we also examined how collectives change in time. To examine the temporal dynamics, we follow collectives in the last 200 timesteps of the evolutionary simulations (T_{track}). At each timestep, we determine the spatial configuration of cells within the collective, which is determined by the spatial coordinates of both adhesive and non-adhesive cells. In total, we analyzed the spatial configuration of $\sim 5 \cdot 10^7$ collectives. we subsequently compared the spatial configurations of these collectives, by correcting for radial symmetry, and counting the number of times each spatial configuration is observed (see also S8 Fig). In total, we observed 402.434 unique spatial configurations. Only 4% of these configurations were observed in all three types of regulation. This low percentage is largely explained by the fact that large collectives are less likely to adopt the same spatial configuration than small collectives, irrespective of the type of regulation, making it unlikely to observe large collectives with the same spatial configuration twice. Indeed, when limiting our analysis to the 3.107 most-observed configurations, expressed by at least 100 of the $5 \cdot 10^7$ collectives, 96% of them are observed in all three types of regulation. In the remainder of this section, we focus on these most-observed configurations.

To examine the spatial configuration of collectives, as well as how collectives change their spatial configuration in time, we make use of a network analysis (S9-S13 Fig). The network gives an overview of all spatial configurations. Each node in the network represents a unique spatial configuration observed in collectives of any type of regulation (S9a Fig). The size of the node shows how many collectives were observed to express a particular spatial configuration. Two nodes are connected, when a collective was observed to change from one spatial configuration to the other in two subsequent timesteps (S11 and S12 Fig). Nodes that are close in network space share many nodes to which they are connected, and nodes that are far apart share few or no nodes to which they are connected (following the Fruchterman-Reingold algorithm [1], S9 and S11 Fig). In this way, the network shows both the diversity of spatial configurations, their similarity and how often each spatial configuration is observed.

The network analysis recapitulates the findings from the main text. First, small collectives are more abundant than large collectives (S9a,b Fig). Seconds, collectives differ in both size and the fraction of adhesive cells (S9b,c Fig). Third, collectives from the different types of regulation express different properties (S10 Fig). In the first type of regulation, where cells have no information about their neighbors, collectives are often small and have a relatively high fraction of adhesive cells. In the second type of regulation, where cells can sense the fraction of adhesive neighbors, collectives have a low fraction of

adhesive cells. In the third type of regulation, where cells can also sense kin, large collectives are more commonly observed.

In addition to these findings, the network analysis reveals that, irrespective of the type of regulation, collectives undergo widely different changes in their spatial configuration in time (S11 and S12 Fig). That is, collectives with the same spatial configuration can change into many different spatial configurations in the next timestep (S12 Fig). Thus, even though regulation gives rise to a reproducible spatial organization of cells within the collective, e.g. central-peripheral polarity in cell adhesion, the exact spatial configuration of cells still varies strongly in time. This corresponds to what we know from surface-associated organisms in nature, where the exact shape and size of an organism often depends on growth opportunities that spontaneously arise on the surface and are not hardwired through a form of deterministic growth and development (such as that observed in form example mammalian development). In contrast to the large diversity in surface growth, collectives show surprisingly similar patterns when it comes to reproduction (S13 Fig). Nearly all reproductive events result in non-adhesive single-cell propagules ($> 90\%$). This shows that fragmentation events, where a collective fragments into one or more smaller collectives on the surface, are relatively rare and that most collectives reproduce by releasing single cells to the bulk. Propagule production is commonly observed among surface-associated organisms in nature, but in contrast to the spontaneous and continuous release of propagules in our simulations, these organisms often tightly control when and where propagules are produced [2,3].

References

1. Fruchterman TMJ, Reingold EM. Graph drawing by force-directed placement. Software: Practice and Experience. 1991;21: 1129–1164. doi:10.1002/spe.4380211102
2. Bonner JT. First signals: the evolution of multicellular development. Princeton: Princeton University Press; 2000.
3. Buss LW. The Evolution of Individuality. Princeton: Princeton University Press; 1987.
